# Carminic acid/ferric ion self assembled multienzyme mimetic nanodrug with concurrent photothermal/anti-inflammatory activity to prevent breast cancer metastasis

**DOI:** 10.1016/j.mtbio.2025.102028

**Published:** 2025-06-28

**Authors:** Mingcheng Wang, Huixi Yi, Qibao Zheng, Muhammad Adnan Younis, Liyou Guo, Zhixiong Zhan, Muhammad Rizwan Younis, Chengzhi Jin, Dong-Yang Zhang

**Affiliations:** a Guangzhou Municipal and Guangdong Provincial Key Laboratory of Molecular Target & Clinical Pharmacology, The NMPA and State Key Laboratory of Respiratory Disease, The Fifth Affiliated Hospital and School of Pharmaceutical Sciences, Guangzhou Medical University, Guangzhou, 511436, PR China; b Department of Chemical and Biomolecular Engineering, University of California-Los Angeles, Los Angeles, CA, 90095, United States; c Institute of Optical Functional Materials for Biomedical Imaging, School of Chemistry and Pharmaceutical Engineering, Shandong First Medical University & Shandong Academy of Medical Science, Taian, Shandong, 271016, PR China

**Keywords:** Self assembly, Multienzyme mimetic, Photothermal therapy, Anti-inflammation, Cancer metastasis

## Abstract

The high metastatic rate of breast tumor is the prominent reason of its poor prognosis, while the inflammatory microenvironment of tumor tissues further promoted tumor metastasis. Although photothermal therapy (PTT) displays high antitumor efficacy, the rise of inflammation-induced reactive oxygen species (ROS) during PTT exacerbate tumor metastasis. To prevent breast tumor metastasis and relieve inflammation-induced oxidative stress during PTT, herein, we developed self-assembled nanodrugs (FCP) consisting of carminic acid, iron ion, and polyvinylpyrrolidone, demonstrating photoacoustic imaging-guided PTT and anti-inflammatory activity to restrict the growth of both primary breast tumor and metastatic tumor. The as designed self-assembled spherical FCP nanoparticles (NPs, 41 nm) exhibited good light to heat conversion and broad-spectrum multienzyme (superoxide dismutase, etc.) mimetic activity to scavenge excess ROS and alleviate oxidative stress. Meanwhile, FCP NPs showed a positive correlation between the photothermal heating and ROS scavenging, allowing the continuous consumption of ROS during PTT. Importantly, owing to the intrinsic bimodal photothermal and photoacoustic imaging abilities, FCP NPs effectively guided and monitored the *in vivo* treatment process, which facilitated to restrict the growth of both primary and metastatic breast tumor *in vivo* due to coordinated PTT and anti-inflammation as confirmed by TUNEL, ki67, and matrix metalloproteinase-9 stainings. Whereas FCP NPs did not pose any potential damage to the vital organs, presenting good biosafety *in vivo*. We envision that self-assembled nanodrugs with concurrent anti-inflammatory and photothermal activities may have great clinical prospects in the treatment of metastatic cancers.

## Highlights

• Self-assembled FCP nanodrug was developed for photothermal tumor therapy.
• FCP nanodrug exhibit multienzyme-like activity for ROS scavenging.
• Photoacoustic/photothermal imaging provide guidance for *in vivo* tumor treatment.
• FCP nanodrugs eliminate primary tumor and further restrict distant tumor metastasis.

## Graphical abstract

**Figure:**
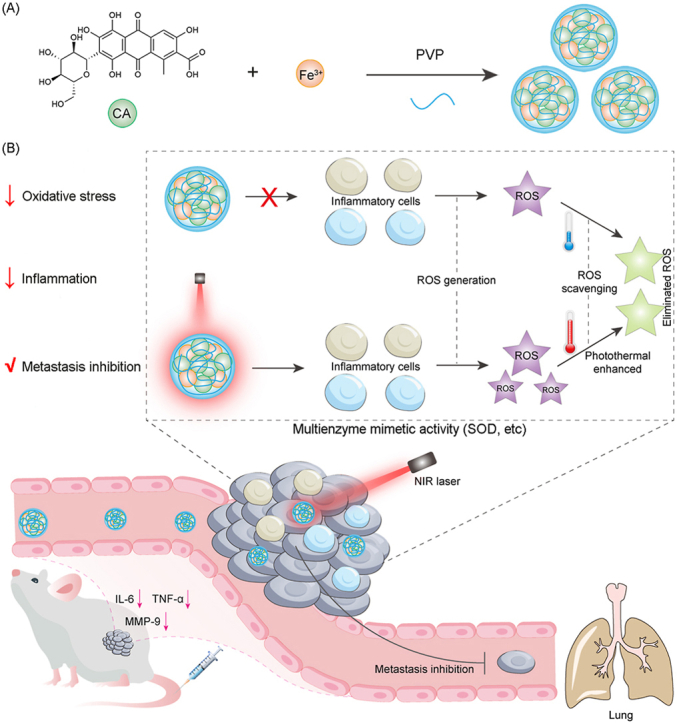

## 1 Introduction

According to the cancer statistics, breast cancer is the leading cancer in women's globally with approximately 2.26 million new breast cancer cases and 680,000 deaths in 2020 [1]. Notably, at the later stages, breast cancer metastasize to the distant organs of the body, which poses serious threats to the women's lives and leads to the high mortality of breast cancer patients [2,3]. After extravasation from the extracellular matrix, breast cancer cells enter into the blood circulation and proceed to the neighboring tissues and vital organs (such as lungs, liver, and bone) through lymphatic vessels and blood vessels, and rapidly proliferate at distant places to develop into fully grown metastatic tumor [[4], [5], [6]]. Patients with distant metastasis at the time of diagnosis have the poorest prognosis, with only 23 % surviving for 5 years after diagnosis. At present, surgical resection, neoadjuvant chemotherapy, and radiotherapy are the available clinical options, but they showed poor treatment efficacy for metastatic breast cancer as they are unable to modulate biological pathways to restrict cancer metastasis [[7], [8], [9]]. Therefore, there is an urgent need to develop safe and effective drugs to treat metastatic breast cancer, prevent distant metastasis, and address the problem of poor prognosis.

The association between cancer and inflammation was first discussed in the 19th century, when Virchow found that tumors were often present at the sites of chronic inflammation, while the infiltration of inflammatory cells was also observed in tumor samples by biopsy [10]. The activation of oncogenes leads to the initiation of multiple transcription factors and the production of inflammation-related cytokines and chemokines, thereby recruiting inflammatory mediators such as macrophages, neutrophils, and myeloid suppressor cells to generate inflammatory microenvironment in breast and other tumors. Further activation of key transcription factors in inflammatory cells and stromal cells to maintain and amplify tumor-related inflammation promote the malignant proliferation of tumors and also provide a microenvironment for vascular infiltration and lymph node diffusion, respectively [[11], [12], [13]]. According to the clinical studies, inflammation was detected in all tumor tissues, which was aggravated in most treatments and induced tumor recurrence and metastasis. Anti-inflammatory drugs showed inhibitory effects on primary tumor growth and metastasis, and post-surgical anti-inflammatory therapy can reduce the probability of post-operative tumor recurrence [14,15]. However, the release kinetics of anti-inflammatory drugs should be controlled to match the degree of inflammation that fluctuates widely during treatment, which otherwise aggravated the side effects due to the excessive inflammatory reactions [16,17]. Therefore, more precise and controllable anti-inflammatory strategies are needed to control cancer metastasis.

Reactive oxygen species (ROS) have been reported to be associated with more than 150 human diseases. Since excessive ROS can promote the development of inflammation-related diseases, reducing tumor ROS level is an effective way to control and modulate inflammation [[18], [19], [20]]. On the other hand, metastasis of tumor cells largely rely on the ability of matrix metalloproteinases (MMPs) to degrade extracellular matrix, thus migrate to the blood or lymphatic vessels. The proliferation of inflammatory cells in the tumor area is accompanied by the production of large amounts of ROS, which can induce the secretion of MMPs from tumor cells and stromal cells, and promote tumor metastasis to the distant organs [[21], [22], [23]]. Therefore, relieving tumor-associated inflammation by scavenging ROS has great potential to inhibit breast cancer metastasis.

Photothermal therapy (PTT) is a spatiotemporally controlled and non-invasive tumor treatment modality, which showed great clinical prospects [[24], [25], [26], [27]]. However, many photothermal agents damage the cell membrane during PTT, which resulted in the leakage of intracellular substances. Such substances trigger the production of ROS, the recruitment and activation of immune cells to release inflammatory factors, such as tumor necrosis factor (TNF-α) and interleukin-6 (IL-6), thus promoting tumor metastasis [[28], [29], [30]]. Therefore, it is necessary to develop photothermal agent with concurrent photothermal and ROS scavenging abilities to ablate cancer cells and prevent ROS production.

**Scheme 1:**
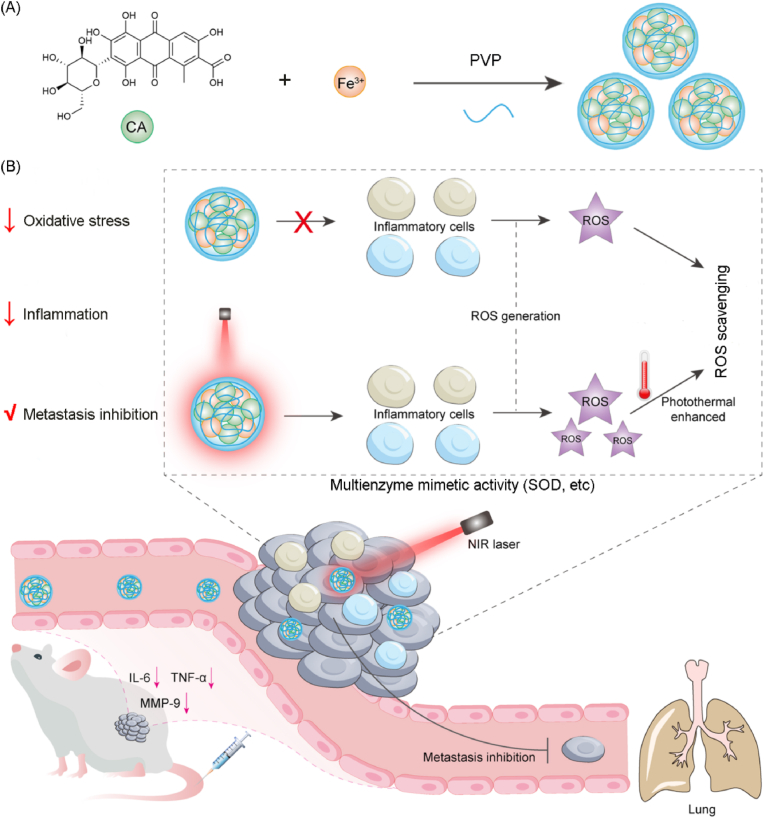
Demonstration of one pot strategy to develop self-assembled nanodrugs, exhibiting multienzyme mimetic activity to relieve oxidative stress and inflammation by scavenging broad-spectrum of ROS, and eliminate primary breast cancer and further restrict cancer metastasis to the distant organs.

Carmine acid is an excellent colorant for food, cosmetics, medicine and textiles. It is also a natural pigment approved by FDA for use in food, medicine, and cosmetics. Due to the ROS scavenging ability, carmine acid exhibits potential anti-tumor properties. In this work, we presented a self-assembly strategy to develop multienzyme mimetic nanodrugs for collaborative PTT/anti-inflammatory therapy of metastatic breast cancer (Scheme 1). In brief, carminic acid, ferric ion, and the capping agent polyvinylpyrrolidone (PVP) were self-assembled to form spherical nanoparticles (NPs), which hold good photothermal conversion ability and photothermal stability. At the same time, the NPs exhibited multienzyme mimetic (superoxide dismutase, etc.) activities to scavenge broad-spectrum ROS and reduce inflammation during PTT treatment. Moreover, the catalytic activity of NPs is positively correlated with the temperature, allowing them to coordinate PTT and ROS mediated anti-inflammation. Therefore, NPs effectivley ablated primary breast cancer in *in situ* 4T1 breast tumor animal model by PTT while the modulation of tumor inflammatory microenvironment and the down-regulation of metastasis-related proteins further restrict breast cancer metastasis to the distant organs. We anticipate that the present work will provide a new insight in combating metastatic breast cancer by modulating the oxidative stress and inflammation.

## 2 Materials and methods

### 2.1 Materials

Carminic acid, 1,1-diphenyl-2-picrylhydrazyl radical (DPPH), methionine, riboflavin, and nitro bluetetrazolium (NBT), and cytochrome C were purchased from Aladdin Reagent (China). Iron trichloride hexahydrate was bought from J&K Scientific (Beijing, China). PVP (average MW55000), 2,2′-azino-bis(3-ethylbenzthiazoline-6-sulfonate) (ABTS), 2′,7′-dichlorofluorescein diacetate (DCF) and thiazolyl blue (MTT) were bought from Sigma-Aldrich (USA). Propidium iodide (PI)/calcein acetoxy-methyl ester (Calcein-AM) and apoptosis detection kit were obtained from Beyotime Biotechnology (China). Matrix metalloproteinase-9 (MMP-9) antibody was purchased from Signalway Antibody (USA). Tumor necrosis factor (TNF-α) and interleukin (IL-6) ELISA kits were obtained from Neobioscience (China). Hydrogen peroxide (H_2_O_2_) and nitric acid were bought from Guangzhou Chemical Reagent Factory (China).

### 2.2 Synthesis of FCP NPs

The FCP NPs was prepared based on previous report and replaced with carminic acid [30]. PVP (80 mL, 8 mg/mL) solution was mixed with 20 mL iron trichloride hexahydrate (8 mg/mL) solution, and stirred for 30 min. Then, 20 mL carminic acid (2 mg/mL) solution was added slowly to the above solution and stirred for 24 h. The solution was then purified by dialysis to obtain NPs, and the obtained samples were freeze-dried.

### 2.3 Photothermal performance evaluation of FCP NPs

Various concentrations of FCP NPs solutions were irradiated with a 808 nm laser for 5 min, and imaged by a thermal imager. The photothermal conversion efficiency of FCP was calculated using the heating/cooling method [[31], [32], [33]]. The photothermal stability of FCP NPs was also evaluated by repeated irradiation (4 times) for 5 min and cooling for 7 min.

### 2.4 Enzyme mimetic activity of FCP NPs

To determine enzyme mimetic activity, we employed superoxide anion assay kit (Sigma-Aldrich, USA) and hydroxyl radical antioxidant capacity (HORAC) assay kit (Cell Biolabs, Inc., USA) to assess superoxide anion and hydroxyl radical scavenging capacity of FCP NPs with indicated concentrations [34], following the manufacturer's protocol. For the experiment of photothermal enhanced superoxide anion removal, the original treatment time of 20 min with reagents was reduced to 2 min, and the corresponding time of light treatment (1 W/cm^2^) was carried out, and then followed the manufacturer's protocol. Meanwhile ABTS and DPPH decolorization tests were also used to detect the scavenging of ABTS and DPPH radicals by FCP NPs with indicated concentrations [35].

The kinetics experiments was performed at 25 °C in PBS (pH 7.4) with FCP (20 μg/mL) in the presence of methionine (12.5 mM), riboflavin (20 μM), FCP NPs (20 μg/mL), and NBT (0–40 μM). The mixtures were illuminated upon UV light with constant intensity at 25 °C for 5 min, and the absorbance change of solutions at 550 nm were monitored. The Michaelis constant (K_m_) was calculated by fitting the Michaelis-Menten equation.

### 2.5 Cytochrome C test

The stock solution of FCP and cytochrome C solution were bubbled with nitrogen for 1 h. Then, FCP NPs (200 μg/mL) were incubated with cytochrome C (400 μM) for 1 h. Subsequently, the absorbance of solution was recorded using a spectrophotometer.

### 2.6 *In vitro* treatment

Various concentrations of FCP NPs (0–400 μg/mL) were incubated with 4T1 cells. After 4 h of incubation, PTT group was irradiated with a near infrared (NIR) laser for 5 min at a power density of 1 W/cm^2^, while the cells from control group were kept in dark (without irradiation). The cells were cultured in the dark for another 24 or 48 h, and the cell viability was quantified by using the standard MTT method [36].

4T1 cells were seeded into 6 cm plates and then treated with/without FCP NPs (200 μg/mL) for 4 h. The cells were irradiated with an 808 nm laser (1 W/cm^2^, 5 min). After incubation for 12 h, the cells were stained using PI/Calcein-AM, or the annexin V-FITC apoptosis detection kit.

### 2.7 JC-1 staining

Mitochondrial function was assessed using the JC-1 probe (Beyotime Biotechnology, China) according to the manufacturer's instructions. 4T1 cells were cultured into the confocal dishes and treated with/without FCP NPs (200 μg/mL) for 4 h. For PTT group, cells were irradiated with an 808 nm laser for 5 min (1 W/cm^2^).

### 2.8 Detection of intracellular ATP levels and caspase-3/7 activity assays

Measurement of adenosine triphosphate (ATP) content and caspase-3/7 activity in cells were carried out using the Cell Titer-Glo kit (Promega, USA) and Caspase-Glo® Assay kit (Promega, USA), respectively, according to the manufacturer's instructions. 4T1 cells were cultured into 96-well plates and treated with/without FCP NPs (200 μg/mL) for 4 h. For PTT group, cells were irradiated with an 808 nm laser for 5 min (1 W/cm^2^).

### 2.9 ROS scavenging *in vitro* with FCP NPs

4T1 cells were pretreated with/without 200 μg/mL of FCP NPs for 4 h. The cells in FCP + NIR group were subjected to a laser treatment (5 min, 1 W/cm^2^). H_2_O_2_ (final concentration is 1 mM) was added in 6-well plates and stimulated for 24 h. Subsequently, the cells were stained with DCF probe, and imaged with a confocal laser microscopy.

### 2.10 *In vitro* migration inhibition test

Wound healing assay: After the 4T1 cells in the 6-well plate were scratched with pipette heads, they were incubated with FCP NPs at different concentrations (0–400 μg/mL) for 24 h, and the cells before and after treatment were photographed with an inverted microscope.

Transwell assay: The migration assay were conducted by using 24-well Transwell® permeable supports (Corning, USA) with 10.13039/100022376FCP NPs (0–400 μg/mL) according to the previous method [37], and the cells were also photographed with an inverted microscope.

### 2.11 Western blotting assay

4T1 cells were incubated with various concentrations of FCP NPs (0–400 μg/mL) for 24 h, and the cells were cleaved to produce the total proteins. Based on previous literature [37], western blotting was used to determine the content of proteins by using corresponding antibody.

### 2.12 ELISA assay

4T1 cells was treated with 200 μg/mL of FCP NPs for 4 h, and then incubated with lipopolysaccharide (LPS, 100 ng/mL) for 1 h. Next, the supernatant was transferred into RAW264.7 macrophages in a 96-well plate and cultured for overnight. Finally, the concentrations of TNF-α or IL-6 in supernatant of RAW264.7 macrophages were determined by the corresponding kits.

### 2.13 *In vivo* biodistribution and imaging of FCP NPs

Photoacoustic (PA) imaging of FCP NPs: The 4T1-tumor bearing mice was intravenously administereted with FCP (200 μL, 4 mg/mL), and the PA images of tumor site were acquired (Vevo, LAZR-X, USA) and quantitatively analyzed at different time points.

Inductively coupled plasma mass spectrometry (ICP-MS) measurements: FCP NPs were administered intravenously into mice (200 μL, 4 mg/mL). After 24 h, the major organs, feces, and urine were harvested and digested by nitric acid, and the content of Fe was determined by ICP-MS (Thermo Scientific, USA). The rate of iron release in urine and feces was calculated by excluding the blank group.

Photothermal imaging of FCP NPs: The mice were intravenously injected with FCP NPs (200 μL, 4 mg/mL), and the thermal images of tumor site were recorded every 2 min for 10 min.

The committee review of animal experiments in Guangzhou Medical University approved the protocol for all animal studies, which were conducted in this study.

### 2.14 *In vivo* PTT of FCP NPs

The 4T1-tumor bearing BALB/c mice were assigned into 4 groups (n = 6): a. Received PBS injection (200 μL), b. Received PBS plus NIR irradiation (200 μL), c. Received FCP injection (200 μL, 4 mg/mL), d. Received FCP injection plus NIR irradiation (200 μL, 4 mg/mL), respectively. The NIR and FCP + NIR groups were irradiated with a 808 nm light source for 10 min at 1 W/cm^2^. The tumor volume of mice was recorded every two days, and photographed and weighed at the end of treatment. The harvested tumor tissues from each group after one day of treatment were stained with ki67/TdT-mediated dUTP Nick-End Labeling (TUNEL)/hematoxylin and eosin (H&E).

### 2.15 *In vivo* metastasis inhibition by FCP

The 4T1-tumor bearing mice were divided into 4 groups (n = 5), which intravenously injected with PBS (200 μL) or FCP NPs (200 μL, 4 mg/mL), and treated with/without NIR. The PBS and FCP groups were in the dark, while the NIR and FCP + NIR groups were illuminated for 10 min (808 nm, 1 W/cm^2^). The tumor specimens were subjected to immunofluorescence staining of dihydroethidium (DHE)/MMP-9, and the IL-6/TNF-α levels in serum were measured at 2nd day post-injection. The lung tissues were collected, photographed, and stained at 40th day post-injection.

### 2.16 *In vivo* biocompatibility of FCP

The fresh blood was acquired through the orbit. The hemolytic test were performed using different concentrations of FCP NPs following previous method [37].

The mice were divided into two groups: 1. FCP NPs and 2. PBS. The body weight of mice was monitored every 3 days. After 30 days, blood and major organs were collected from the euthanized mice. The hematologic analysis and liver/kidney markers of mice were measured. Meanwhile, the major organ sections were stained with H&E.

### 2.17 Statistical analysis

Data were presented as means ± standard deviations. The significant differences of all the data were analyzed by Origin 8.5 software according to a student's *t*-test: ∗p < 0.05, ∗∗p < 0.01 and ∗∗∗p < 0.001.

## 3 Results and discussion

### 3.1 Synthesis and characterization

**Fig. 1:**
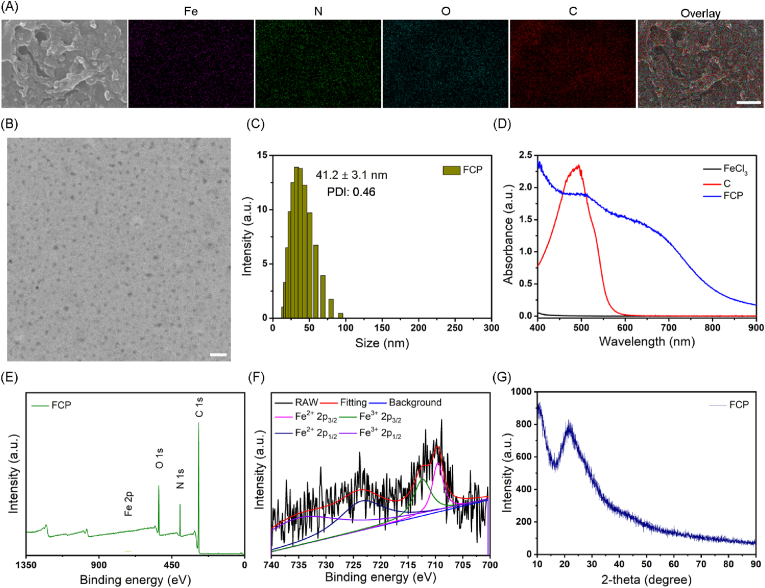
(A) SEM image of FCP NPs and the corresponding elemental mapping. The scale bar is 500 nm. (B) TEM image of FCP NPs. The scale bar is 200 nm. (C) Hydrodynamic diameter of FCP NPs as measured by DLS. (D) Absorption spectra of FeCl_3_, carminic acid: C, and FCP solutions. (E) XPS survey scan and (F) Fe *2p* XPS spectra of FCP NPs. (G) XRD pattern of FCP samples.

Iron ions, PVP, and carmine acid were self-assembled at a room temperature to obtain spherical FCP nanoparticles. Elemental mapping confirmed the presence of Fe, C, O, and N elements in the as-obtained FCP NPs, suggesting that FCP is composed of Fe, carmine acid, and PVP (Fig. 1A and S1). FCP NPs were about 30–40 nm in diameter and showed good dispersibility as characterized by transmission electron microscope (TEM, Fig. 1B). Meanwhile, the hydrodynamic diameter and polydispersity index of FCP NPs was 41.2 ± 3.1 nm and 0.46, as detected by dynamic light scattering (DLS, Fig. 1C). While, the nanoparticles displayed good stability in different physiological solutions for a long time (Fig. S2). Compared with the free iron ion and carmine acid, the self-assembled FCP NPs have an obvious absorption in the near infrared (NIR, Fig. 1D) region, with an absorption coefficient of 23.7 L/(m × g) at 808 nm (Fig. S3). Furthermore, X-ray photoelectron spectroscopy (XPS) was carried out to analyze the component of FCP. As shown in Fig. 1E, the characteristic peaks of N *1s* (399.4 eV) and Fe *2p* (711.2 eV) were found, confirming that FCP NPs are composed of Fe ions and PVP. While, Fe *2p* XPS spectra indicated the two valence states of Fe element (709.5 eV, 712.5 eV, 722.4 eV, 735.3 eV were assigned to Fe^2+^ *2p*_*3/2*_, Fe^3+^ *2p*_*3/2*_, Fe^2+^ *2p*_*5/2*_, and Fe^3+^ *2p*_*3/2*_, respectively) in FCP NPs (Fig. 1F), which endow FCP NPs with multienzyme mimetic activity [38,39]. Besides, the X-ray diffraction (XRD) spectrum indicated the amorphous nature of FCP NPs as shown in Fig. 1G. The morphology of FCP NPs undergoes significant changes in an acidic solution (pH 5.0) and the hydrodynamic diameter reduces to 22.3 nm (Fig. S4), indicating the degradation of FCP NPs under acidic conditions.

### 3.2 Photothermal performance and ROS scavenging

**Fig. 2:**
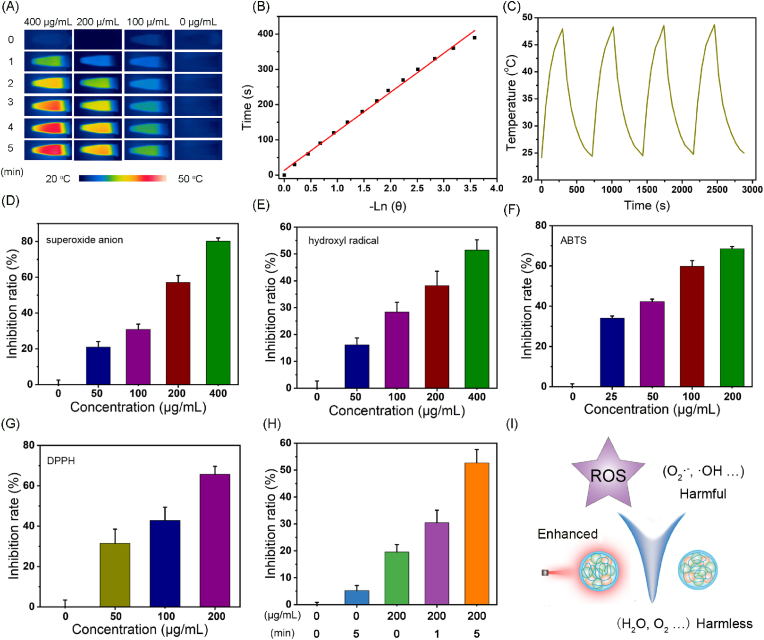
(A) Digital images of real-time photothermal enhancement by FCP NPs under NIR laser irradiation. (B) Plot of negative natural logarithm versus cooling time of the temperature driving force, which was obtained from the cooling stage. (C) Photothermal stability of FCP NPs was detected under sequential NIR laser irradiation. Evaluation of (D) superoxide anion, (E) hydroxyl radical, (F) ABTS, and (G) DPPH scavenging ability of FCP NPs. (H) Superoxide anion scavenging by FCP NPs with different NIR laser illumination times. (I) Schematic diagram of photothermal enhanced broad spectrum ROS scavenging by FCP NPs.

Next, we determined the light to heat conversion ability of FCP NPs. A significant increase in solution temperature was recorded with an increase in the exposure time (0–5 min) and the concentration of NPs (0–400 μg/mL), suggesting time-dependent and concentration-dependent photothermal activity (Fig. 2A and S5). The temperature of the solution at 400 μg/mL increased by approximately 23 °C, while ∼2 °C temperature change was recorded in the control group. Subsequently, the photothermal conversion efficiency of FCP NPs was calculated to be 20 % following the heating/cooling method (Fig. 2B and S6). Under sequential laser on/off cycles, there was no noticeable change in the photothermal effect (Fig. 2C). Whereas, TEM characterization after 5 consecutive laser on/off cycles showed the intact morphology without any degradation or aggregation (Fig. S7), which suggested the good photothermal stability of FCP NPs. Considering the presence of phenol hydroxyl groups and other structures, we also assessed the ROS scavenging activity of FCP. As shown in Fig. 2D and E, FCP NPs (400 μg/mL) scavenged about 80 % and 51 % of superoxide anions and hydroxyl radicals as measured by the relevant assay kits. Furthermore, the K_m_ value and maximum reaction rate (V_max_) of FCP NPs with SOD-like activity were about 15.1 mM and 0.075 μM/min as determined by Michaelis-Menten equation (Fig. S8). In addition, ABTS and DPPH free radicals were chose to appraise whether FCP NPs can scavenge broad-spectrum ROS. As presented in Fig. 2F and G, FCP NPs scavenged ABTS and DPPH in a concentration-dependent manner, proving the scavenging of a broad spectrum of ROS. At 200 μg/mL concentration, the scavenging efficiency was roughly 69 % and 68 % for ABTS and DPPH. It is notable to mention that the catalytic ROS scavenging ability of FCP NPs was positively correlated with the laser exposure time as shown in Fig. 2H. In dark conditions, FCP NPs displayed 5.2 % scavenging of superoxide anions, whereas under NIR laser irradiation for 5 min, more than 50 % superoxide anions were eliminated, suggesting their potential to coordinate between PTT and ROS scavenging (Fig. 2I) [28,40,41]. The potential ROS scavenging mechanism of FCP was analyzed through cytochrome C assay. As shown in Fig. S9, the reduced form of cytochrome C showed two characteristic absorption peaks at 520 and 550 nm. However, after co-incubation of FCP NPs with nitrogen or air, both the peaks disappeared and a new peak emerged at 530 nm, indicating that cytochrome C can transfer electrons to FCP, and this process is independent of oxygen.

### 3.3 *In vitro* treatment

**Fig. 3:**
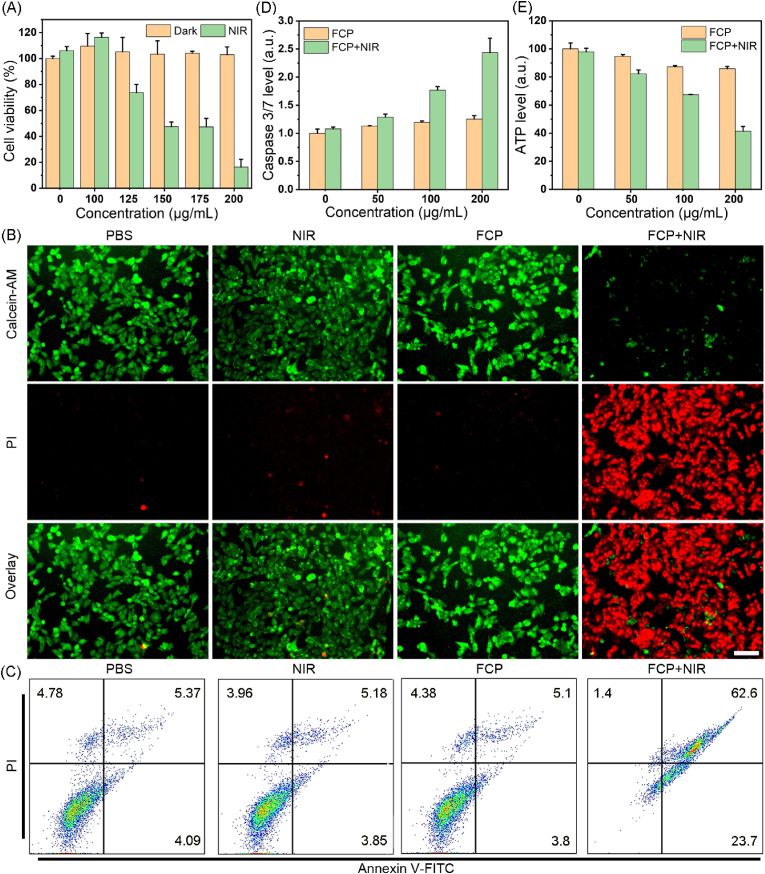
(A) Cell viability of 4T1 cells under different conditions as monitored by MTT assay. (B) Live/dead staining of 4T1 cells treated with PBS, FCP NPs in the dark, and FCP NPs under NIR laser excitation as mentioned. Scale bar: 100 μm. (C) Apoptosis of 4T1 cells undergo different treatments. (D) Quantitative analysis of ATP and (E) caspase 3/7 levels in 4T1 cells under different conditions.

Since FCP NPs exhibit good photothermal activity and stability, we assessed the *in vitro* photothermal therapeutic performance. The MTT assay was first performed to test the therapeutic performance of FCP NPs using breast cancer (4T1) cells. Under dark condition, there was no noticeable toxicity on 4T1 and human embryonic kidney (HEK) 293T cells even at any concentrations (Fig. 3A and S10). In contrast, under laser excitation, the PTT group showed significant inhibition of breast cancer cells. At the concentration of 200 μg/mL, the cell survival rate was more than 90 % in the dark condition, while the cell survival rate after photothermal treatment by FCP NPs drops to 10 %. As the incubation time extended to 48 h, FCP (200 μg/mL) showed inhibitory effects on the 4T1 cells with a inhibition rate of about 33 % (Fig. S11). Similarly, live and dead staining and apoptosis detection also indicated good photothermal therapeutic effects of FCP NPs on breast cancer cells (Fig. 3B and C) as the highest percentage of dead cells and apoptosis were recorded after PTT treatment. Notably, FCP NPs (200 μg/mL) + NIR treatment significantly reduced the proportion of green fluorescence of the JC-1 probe, suggesting that FCP NPs disrupted the mitochondrial function under laser activation (Fig. S12 and S13). At the same time, it decreased the intracellular ATP level to 41 %, and through the use of a kit, it also elevated the apoptosis-related proteins caspase3/7 by 2.4 times (Fig. 3D and E). This confirmed that under laser activation, the FCP NPs induced cell apoptosis [[35], [36], [37]].

### 3.4 *In vitro* migration inhibition

**Fig. 4:**
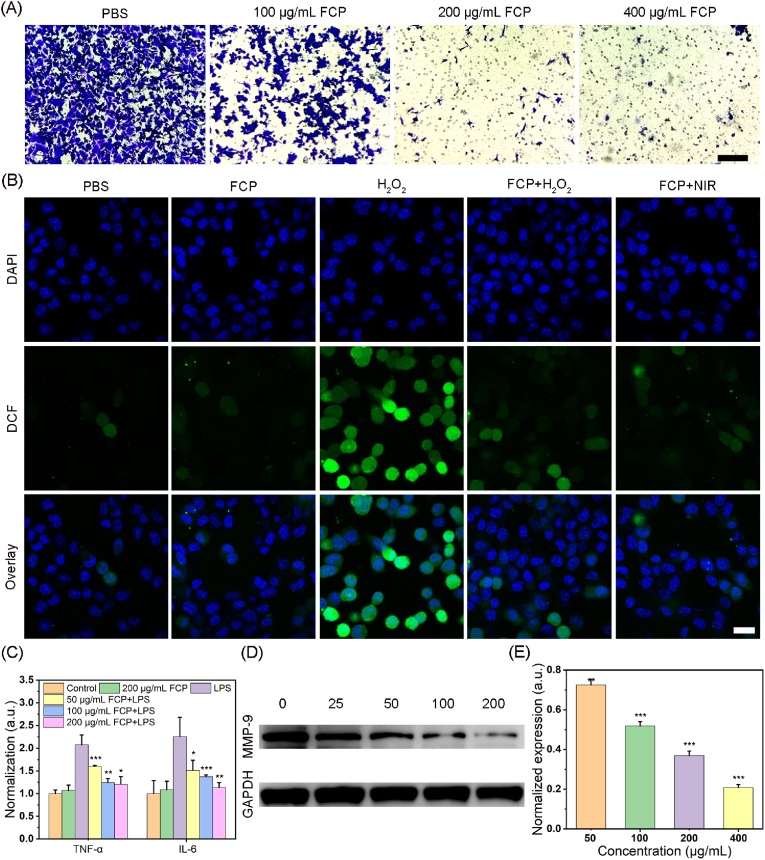
(A) Representative photos of transwell assay under different conditions. Scale bar: 200 μm. (B) Fluorescence images of H_2_O_2_ stimulated 4T1 cells stained with DCF probe after pre-treatment with/without FCP NPs. Scale bar: 20 μm. (C) The level of IL-6 and TNF-α in cell culture supernatant incubated with/without LPS and pre-treated with/without FCP NPs. Data were presented as means ± standard deviations. ∗indicates P < 0.05, ∗∗indicates P < 0.01 and ∗∗∗indicates P < 0.001 vs LPS group. (D) Study the expression of MMP-9 in 4T1 cells under different concentrations (unit: μg/mL) of FCP NPs by western blotting and (E) the corresponding quantitative analysis. Data were presented as means ± standard deviations. ∗indicates P < 0.05, ∗∗indicates P < 0.01 and ∗∗∗indicates P < 0.001 vs control group.

Since cancer metastasis is one of the root causes of its poor prognosis, we performed transwell and wound healing experiments to evaluate the effect of nanomedicine on tumor migration. Representative pictures in Fig. 4A displayed that the number of cell migrations markedly reduced with an increase in the concentration of nanodrug. Quantitative analysis verified nearly 99 % inhibition of breast tumor cells in the transwell assay when treated with FCP NPs at 400 μg/mL (Fig. S14), suggesting that FCP NPs had an impressive tumor migration suppression effect. Analogously, in wound healing measurement, the distinct reduction of the migratory cells was observed with a 93 % inhibition rate (Figs. S15–16), which matched with the results of the transwell assay. Subsequently, the mechanism of action was investigated. Firstly, the levels of ROS in 4T1 cells stimulated by H_2_O_2_ from pretreated NPs were significantly lower than those of unpretreated cells (Fig. 4B). Moreover, the intracellular ROS level in the cells treated with FCP + NIR group did not show a significant increase in the intracellular ROS level as determined by confocal microscopy and flow cytometry (Fig. 4B and Fig. S17), suggesting that FCP NPs possess good photothermal-enhanced ROS scavenging ability. Furthermore, the LPS was used to stimulate macrophages to mimic the inflammatory environment. The LPS-stimulated macrophage cells after pretreatment of NPs exhibited apparently lower secretion of pro-inflammatory factors, such as IL-6 and TNF-α, than untreated cells (Fig. 4C), suggesting that FCP NPs hold anti-inflammatory effects by clearing ROS [[42], [43], [44]]. Since MMP-9 protein regulates angiogenesis, metastasis, and invasion of tumor cells [[45], [46], [47]], the expression in cells after drug treatment were measured by western blot assay. As expected, nanodrugs down-regulate the expression of MMP-9 (Fig. 4D and E), which revealed the inhibitory mechanism of tumor metastasis.

### 3.5 *In vivo* biodistribution and imaging

**Fig. 5:**
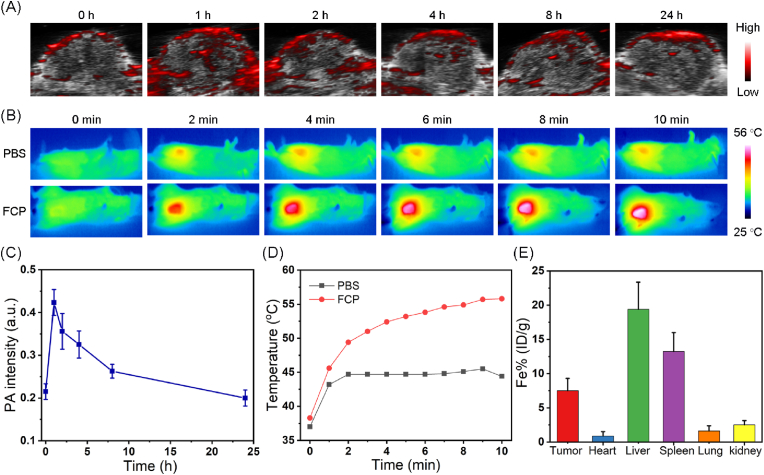
(A) PA imaging *in vivo* at different time points after intravenous injection of FCP NPs and (C) the corresponding quantitative analysis. (B) Photothermal imaging *in vivo* by FCP NPs under laser irradiation for different time points and (D) the corresponding quantitative analysis. (E) *In vivo* biodistribution of FCP NPs at 24 h post-injection by ICP-MS analysis.

Considering the photothermal performance of FCP NPs, the PA effect was evaluated in solution. As shown in Fig. S18, the PA intensity was linearly correlated with the concentration of FCP NPs, implying their potential for PA imaging *in vivo*. Then, the FCP NPs were intravenously administered into 4T1-bearing mice, and the PA signals were collected at different time points within 24 h. As illustrated in Fig. 5A and C, the PA signals of tumor tissues were enhanced after injection of nanodrug. At 1 h post-injection, we recorded the maximum PA signal at tumor area than the later time points (2 h, 4 h, 8 h, 24 h). Subsequently, the time point for laser excitation was ascertained based on the guidance of PA imaging, and the temperature changes at tumor tissue of mice were recorded at 1 h post-injection. The FCP NPs-treated mice exhibited localized temperature elevation of about 17.5 °C in 10 min under irradiation, which was much higher than the PBS-treated group (7.4 °C) as presented in Fig. 5B and D. Whereas, at 24 h post-injection, 6.5 % FCP NPs were found in the tumor region as confirmed by the ICP-MS analysis (Fig. 5E). Furthermore, at 24 h post-injection, the Fe content content in feces and urine was about 32 % and 4.8 % as shown in Fig. S19, which suggest that FCP NPs may be metabolized through the kidneys and liver, respectively.

### 3.6 *In vivo* treatment

**Fig. 6:**
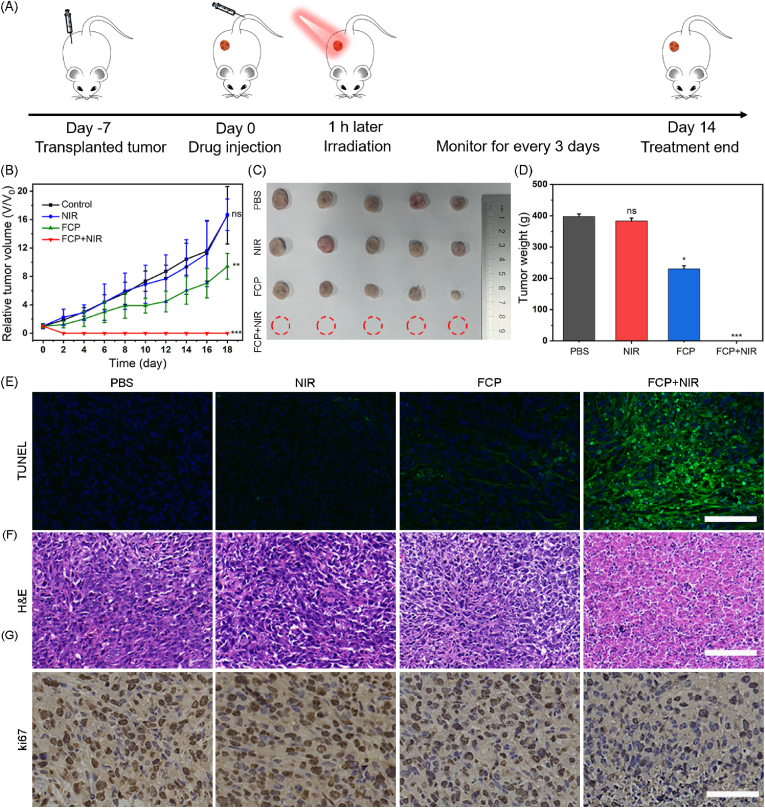
(A) Schematic layout of the *in vivo* treatment process. Relative tumor volume (B), digital photographs of dissected tumors (C), and tumor weight under different conditions (D) as mentioned. (E) TUNEL, (F) H&E and (G) ki67 staining of tumor tissues harvested from different groups. The scale bars are 100 μm, 100 μm, and 50 μm in (E), (F), and (G), respectively. Data were presented as means ± standard deviations. ^ns^indicates P > 0.05, ∗indicates P < 0.05, ∗∗indicates P < 0.01 and ∗∗∗indicates P < 0.001 vs PBS-treated mice.

The promising *in vitro* effect of FCP NPs in killing breast cancer cells through PTT inspired us to study FCP NPs as photothermal agents for concurrent photothermal/anti-inflammatory tumor treatment *in vivo*. 4T1-tumor bearing mice were then randomly divided into four groups (PBS, NIR, FCP, and FCP + NIR). The body weight and tumor volumes of mice from each group were recorded every two days. As presented in Fig. 6A–C, tumor growth curves, digital photographs of dissected tumor, and tumor weights confirmed that FCP NPs in dark could partially inhibit tumor growth than PBS and NIR treatment. While under laser activation, FCP NPs completely eliminated breast cancer. After 2-weeks, tumor tissues were harvested from all groups and performed TUNEL, H&E, and ki67 staining. In Fig. 6D, TUNEL staining exhibited weak green fluorescence in FCP NPs group, which indicated partial killing of tumor cells, In contrast, FCP + NIR group showed effective tumor suppression as confirmed by the significant proportion of intense green fluorescence, which is further verified by H&E staining as shown in Fig. 6E. In addition, compared to the control groups, a noticeable increase in the positive proportion of ki67 staining was seen in FCP and FCP + NIR groups (Fig. 6F), suggesting that the proliferation of 4T1 tumor is significantly reduced. These results demonstrated that FCP NPs under laser activation could completely inhibit the *in situ* breast cancer.

### 3.7 *In vivo* metastasis inhibition

**Fig. 7:**
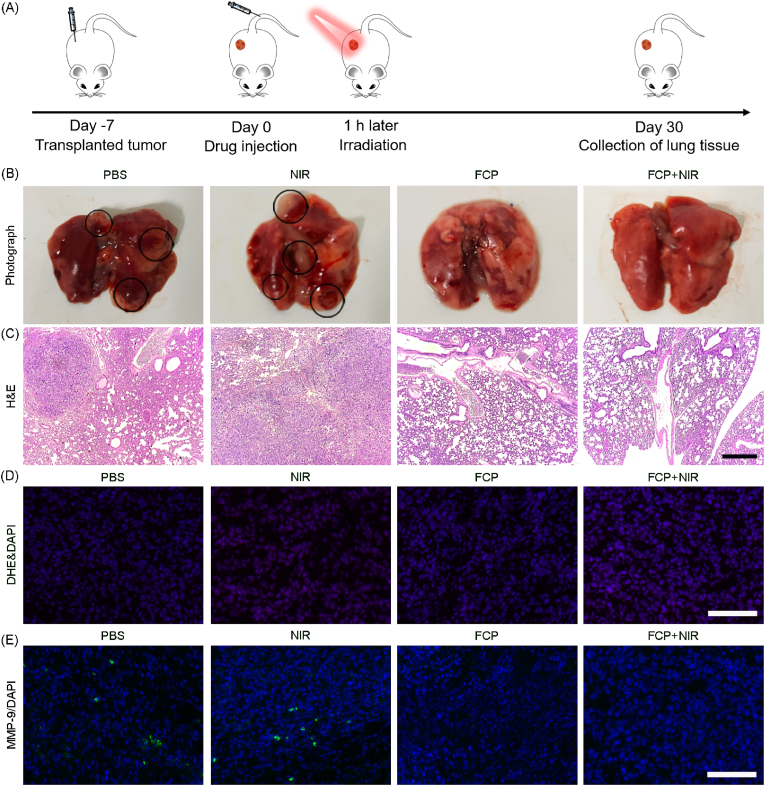
(A) Schematic layout of the *in vivo* treatment process. (B) Digital photographs of *ex vivo* lung samples and circled metastatic tumors region after various treatments. (C) H&E staining of lung tissues under different conditions. Scale bar: 200 μm. (D) DHE stained images of tumor tissues with various treatment. Scale bar: 100 μm. (E) Immunohistochemical staining of tumor tissues by MMP-9 antibody with various treatments. Scale bar: 100 μm.

To study whether FCP NPs can inhibit breast tumor metastasis, tumor metastasis model mice were constructed and the above-mentioned four treatments were carried out. The lung tissues were harvested from different experimental groups 36th days later. The digital photographs indicated an obvious breast cancer metastasis in PBS and NIR groups, whereas both FCP NPs in dark and NIR activated FCP NPs effectively restricted tumor metastasis as shown in Fig. 7A. Besides, H&E staining did not show any noticeable abnormality in both FCP and FCP + NIR groups (Fig. 7B), confirming the suppression of metastatic 4T1 tumor. Considering that PTT-induced superfluous ROS may exacerbate tumor metastasis, DHE staining was also performed to assess ROS level in tumor tissues after different treatments. No noticeable difference was found in the fluorescence intensity of DHE among four treatment groups, which suggested good ROS scavenging *in vivo* before and after photothermal-induced by FCP NPs under NIR laser activation (Fig. 7C). Meanwhile, representative immunohistochemical stained images showed that the expression of MMP-9, which is important for angiogenesis and tumor metastasis, was visibly decreased in the tumor tissues after treated with FCP NPs (Fig. 7D). To verify whether FCP NPs could relieve the inflammatory response induced by PTT, the levels of inflammatory factors in the blood were examined. As seen in Figs. S20–21, there was no significant difference in the serum levels of pro-inflammatory factors (IL-6 and TNF-α) among four treatment groups. These evidences explained and validated that FCP could inhibit breast cancer metastasis through synergistic PTT and ROS scavenging mediated anti-inflammatory therapy.

### 3.8 Biocompatibility assessment

**Fig. 8:**
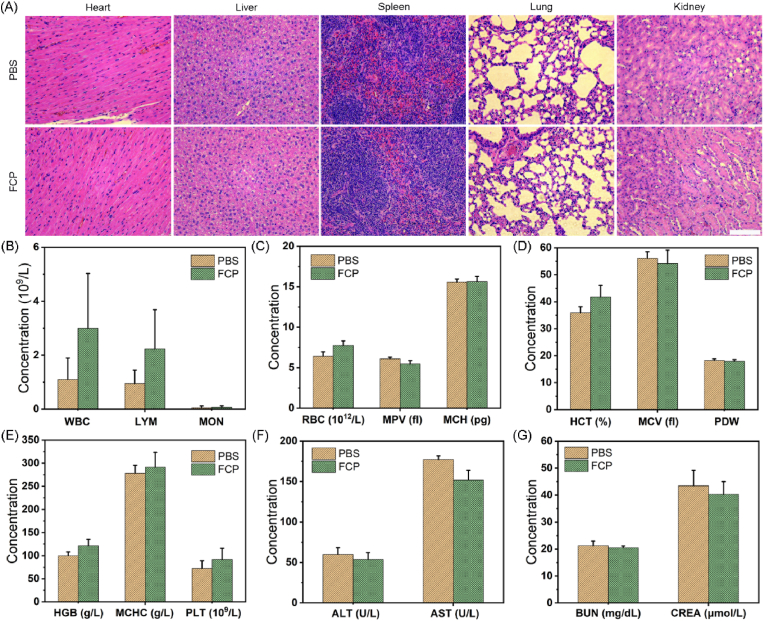
(A) H&E stained images of major organ harvested from PBS and FCP groups after 30 days. Scale bar: 100 μm. (B–E) Whole blood analysis of mice from PBS and FCP groups. The levels of (F) ALT/AST and (G) BUN/CREA in serum after different treatments.

The biological safety of nanomaterials plays a decisive role in their clinical application, so the *in vivo* safety of FCP NPs was evaluated. The impact of various concentrations of FCP NPs solution on red blood cells (RBCs) was first studied. Hemolysis assay indicated less than 5 % damage to the RBCs at any used concentrations of FCP (Fig. S22), suggesting the favourable biocompatibility of FCP NPs. In addition, the organs and blood were harvested from mice treated with PBS or FCP NPs for histological staining and hematological analysis. After intravenous injection of FCP NPs, compared with the mice in PBS group, neither any significant difference in the body weight (Fig. S23) nor any damage/inflammation was observed in the major organs (Fig. 8A), indicating the negligible systemic toxicity. Simultaneously, the prime blood biochemistry indexes of mice with FCP treatment were in a reasonable range (Fig. B–E), revealing the great biocompatibility of FCP NPs. Besides, the liver/kidney biochemical indicators, including alanine transaminase (ALT)/aspartate transaminase (AST) and blood urea nitrogen (BUN)/creatinine (CREA), of mice were in a normal range as compared to the control group as presented in Fig. 8F and G. These findings demonstrated that FCP NPs hold good biological safety and biocompatibility.

## 4 Conclusion

In summary, we employed a self assembly approach to design FCP NPs for coordinating anti-inflammation and PTT to treat metastatic breast cancer. By virtue of good photothermal conversion performance and photothermal stability, FCP NPs triggered irreversible photothermal killing of breast cancer cells. Whereas, multienzyme mimetic characteristics allowed FCP NPs to scavenge a broad range of ROS to alleviate inflammatory response induced by PTT. Under bimodal PA/PT imaging guidance, FCP NPs completely inhibited the tumor growth in 4T1-tumor bearing mice through PTT. Further, they also restricted breast cancer metastasis to the distant organs due to the antioxidant activity as evidenced by the down-regulation of MMP-9 and other inflammatory markers, respectively. We anticipate that self-assembled nanosystems with good coordination between PTT and anti-inflammation will be a potential candidate to treat metastatic breast tumor.

## CRediT authorship contribution statement

**Mingcheng Wang:** Writing – original draft, Visualization, Validation, Methodology, Investigation, Formal analysis, Data curation, Conceptualization. **Huixi Yi:** Writing – original draft, Visualization, Methodology, Investigation, Formal analysis, Conceptualization. **Qibao Zheng:** Visualization, Validation, Software, Methodology, Investigation. **Muhammad Adnan Younis:** Visualization, Validation, Methodology, Investigation, Formal analysis, Data curation. **Liyou Guo:** Writing – original draft, Methodology, Investigation, Formal analysis, Data curation, Conceptualization. **Zhixiong Zhan:** Writing – review & editing, Validation, Software, Methodology, Investigation. **Muhammad Rizwan Younis:** Writing – review & editing, Supervision, Project administration, Funding acquisition, Conceptualization. **Chengzhi Jin:** Writing – review & editing, Validation, Supervision, Project administration, Formal analysis. **Dong-Yang Zhang:** Writing – review & editing, Supervision, Project administration, Funding acquisition, Conceptualization.

## Declaration of competing interest

The authors declare that they have no known competing financial interests or personal relationships that could have appeared to influence the work reported in this paper.

## Data Availability

Data will be made available on request.
