# Supplementary material for: Carminic acid/ferric ion self assembled multienzyme mimetic nanodrug with concurrent photothermal/anti-inflammatory activity to prevent breast cancer metastasis
Source: Mater Today Bio. 2025 Jun 28;33:102028. doi: 10.1016/j.mtbio.2025.102028 (PMC12270016; doi:10.1016/j.mtbio.2025.102028)
Supplement: Multimedia component 1 [file mmc1.docx]

**Supporting information**

Carminic acid/ferric ion self assembled multienzyme mimetic nanodrug with concurrent photothermal/anti-inflammatory activity to prevent breast cancer metastasis

Mingcheng Wang^1,a^, Huixi Yi^1,a^, Qibao Zheng^a^, Muhammad Adnan Younis^b^, Liyou Guo^a^, Zhixiong Zhan^a^, Muhammad Rizwan Younis*^,b,c^, Chengzhi Jin*^,a^, Dong-Yang Zhang*^,a^

1. Guangzhou Municipal and Guangdong Provincial Key Laboratory of Molecular Target & Clinical Pharmacology, the NMPA and State Key Laboratory of Respiratory Disease, the Fifth Affiliated Hospital and School of Pharmaceutical Sciences, Guangzhou Medical University, Guangzhou 511436, China
2. Department of Chemical and Biomolecular Engineering, University of California - Los Angeles, Los Angeles, California 90095, United States
3. Institute of Optical Functional Materials for Biomedical Imaging, School of Chemistry and Pharmaceutical Engineering, Shandong First Medical University & Shandong Academy of Medical Science, Taian, Shandong 271016, China

Corresponding Authors: ryounis@ucla.edu; chengzhijin@gzhmu.edu.cn; [zhangdy7@gzhmu.edu.cn](mailto:zhangdy7@gzhmu.edu.cn).

M. W., and H. Yi. contributed equally to this work.

Supplementary Figures


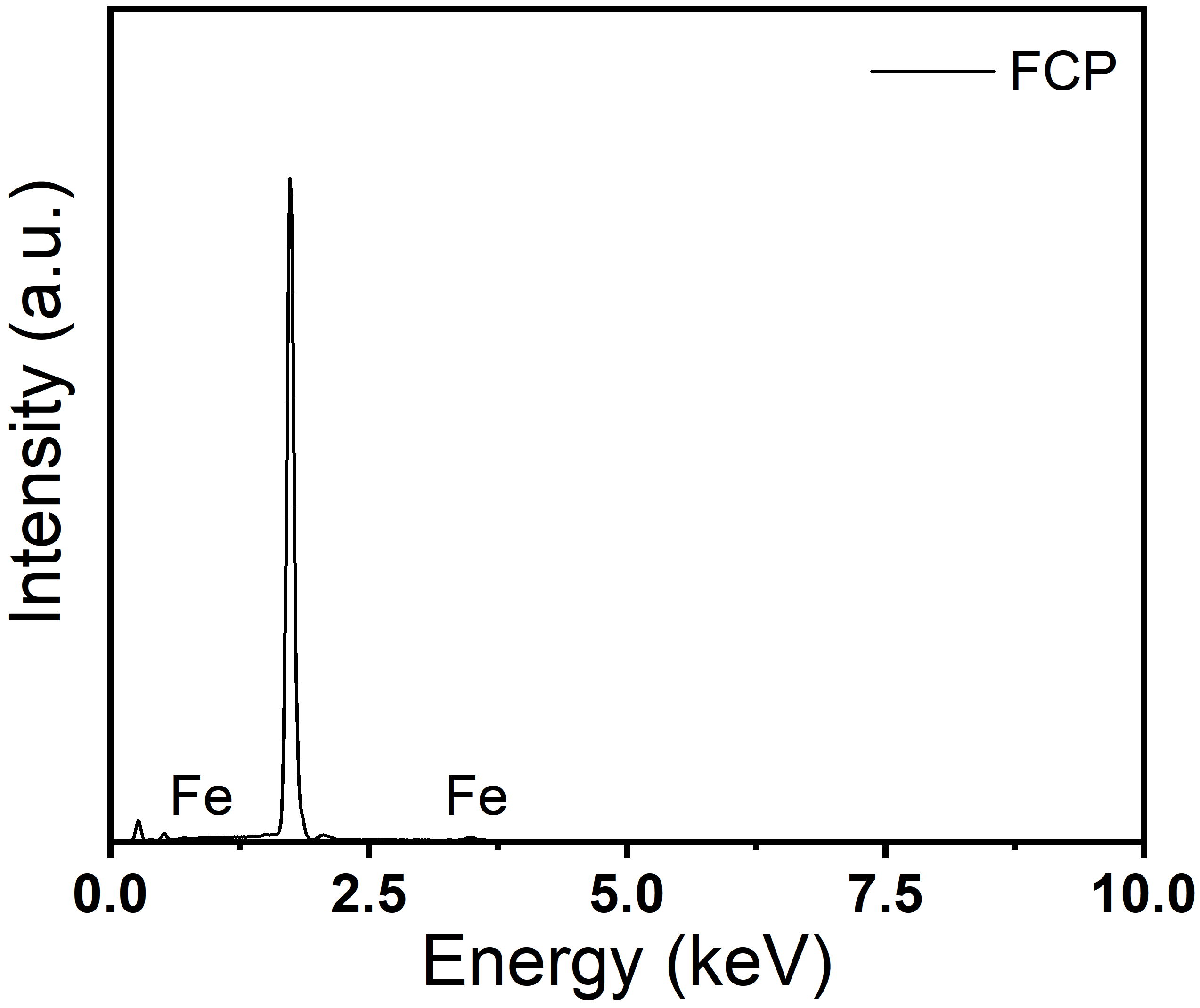


Fig. S1. Energy-dispersive spectrum of FCP NPs.


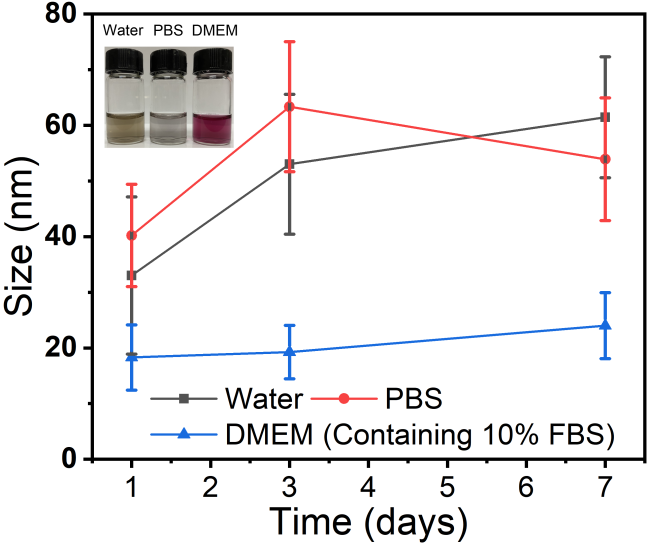


Fig. S2. Hydrodynamic diameter of FCP NPs in different solutions within 7 days. Digital photographs of FCP NPs dispersions in different solvents.


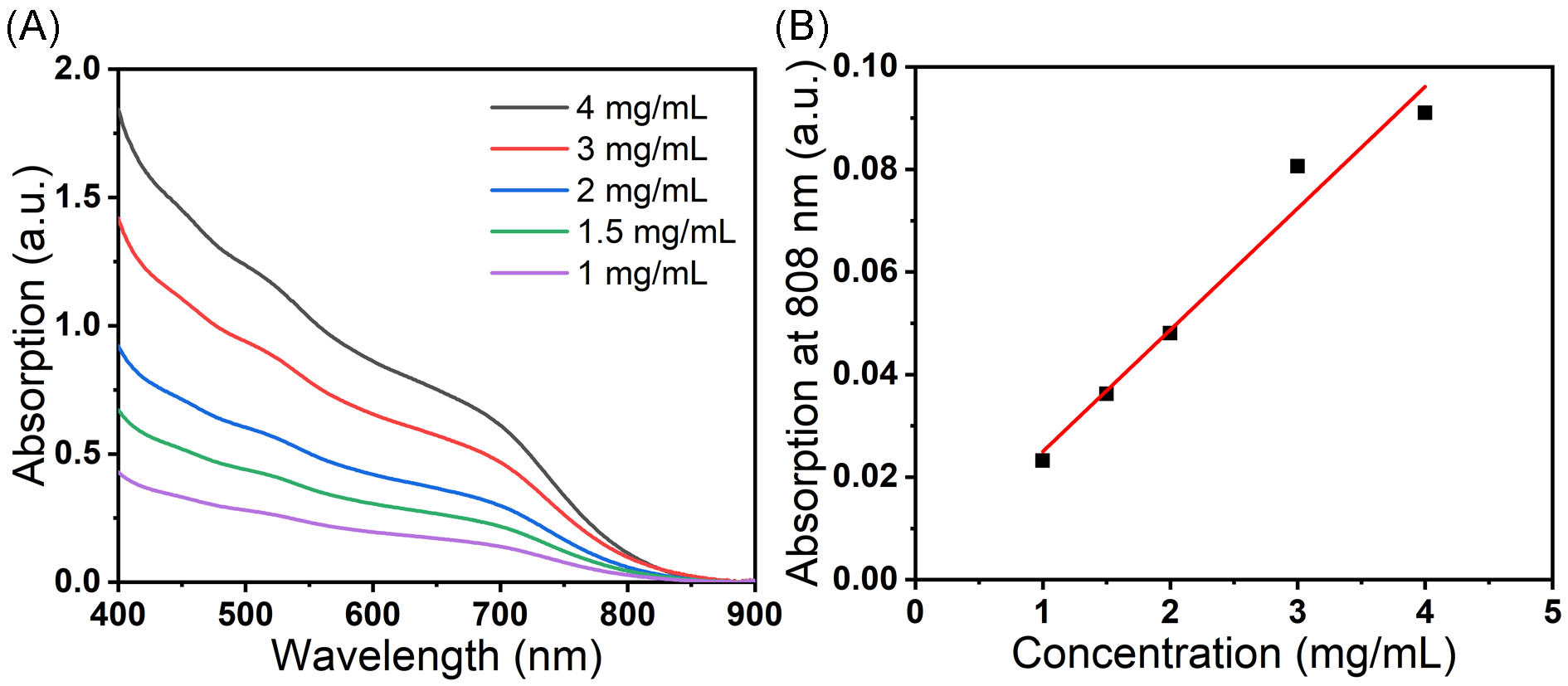


Fig. S3. [Absorption spectra](javascript:;) and optical density value at 808 nm of FCP NPs solution with various concentrations.


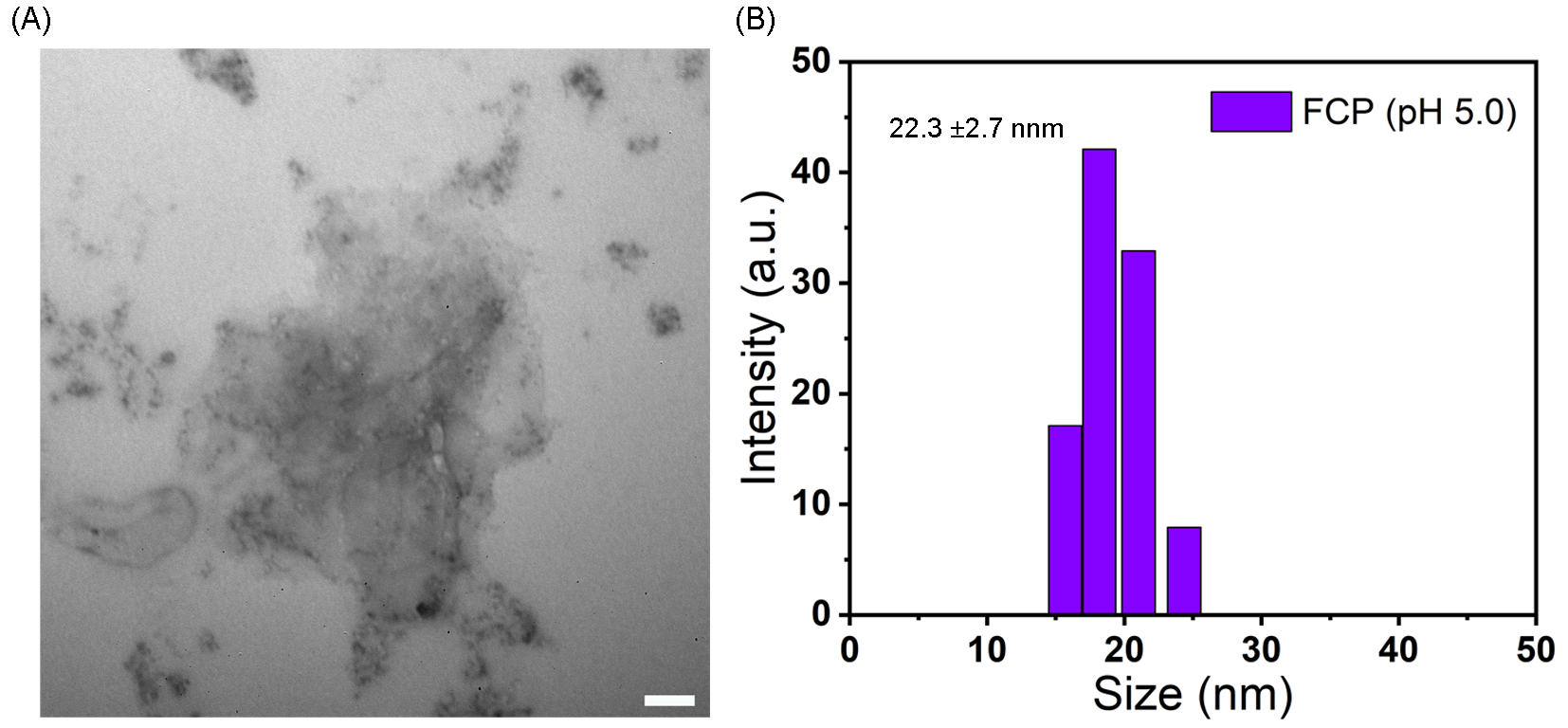


Fig. S4. (A) TEM image and (B) hydrodynamic diameter of FCP NPs in solution at pH 5.0. Scale bar: 100 nm.


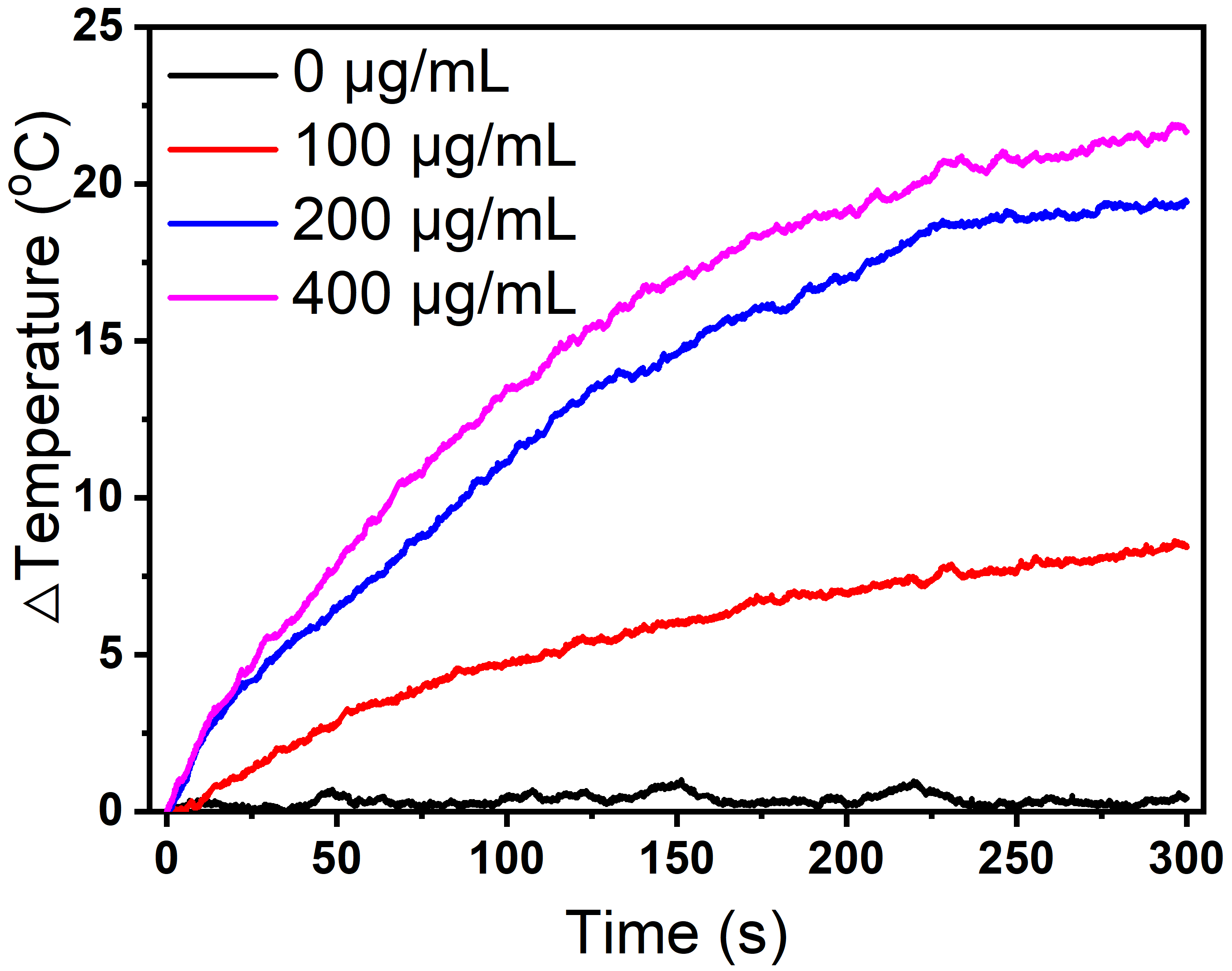


Fig. S5. Temperature curves of FCP NPs solution at different concentrations under 808 nm laser irradiation.


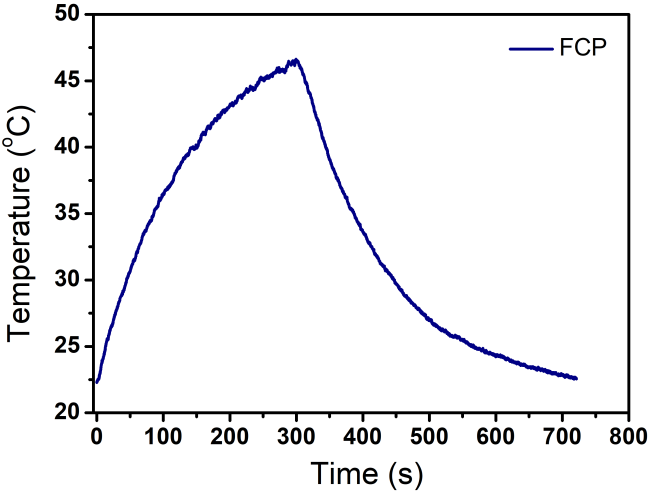


Fig. S6. Temperature curves of FCP NPs solution under 808 nm laser irradiation for 5 min.


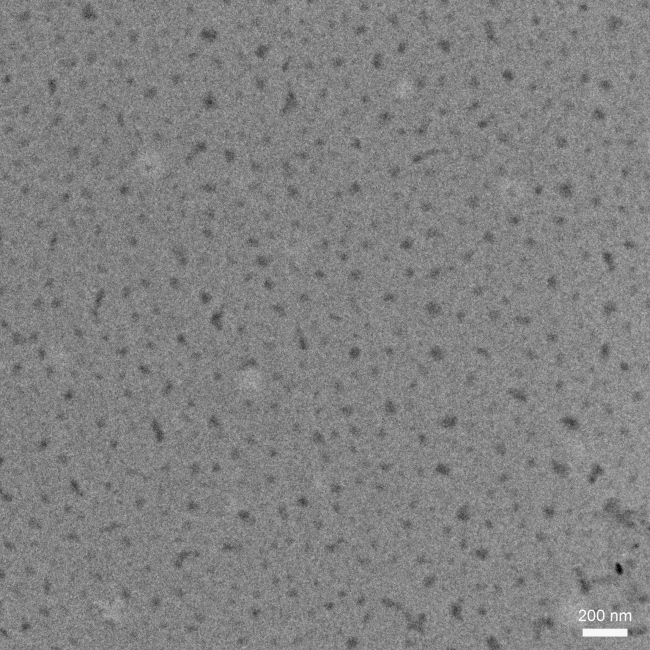


Fig. S7. TEM image of FCP NPs after repeated laser irradiation. Scale bar: 200 nm.


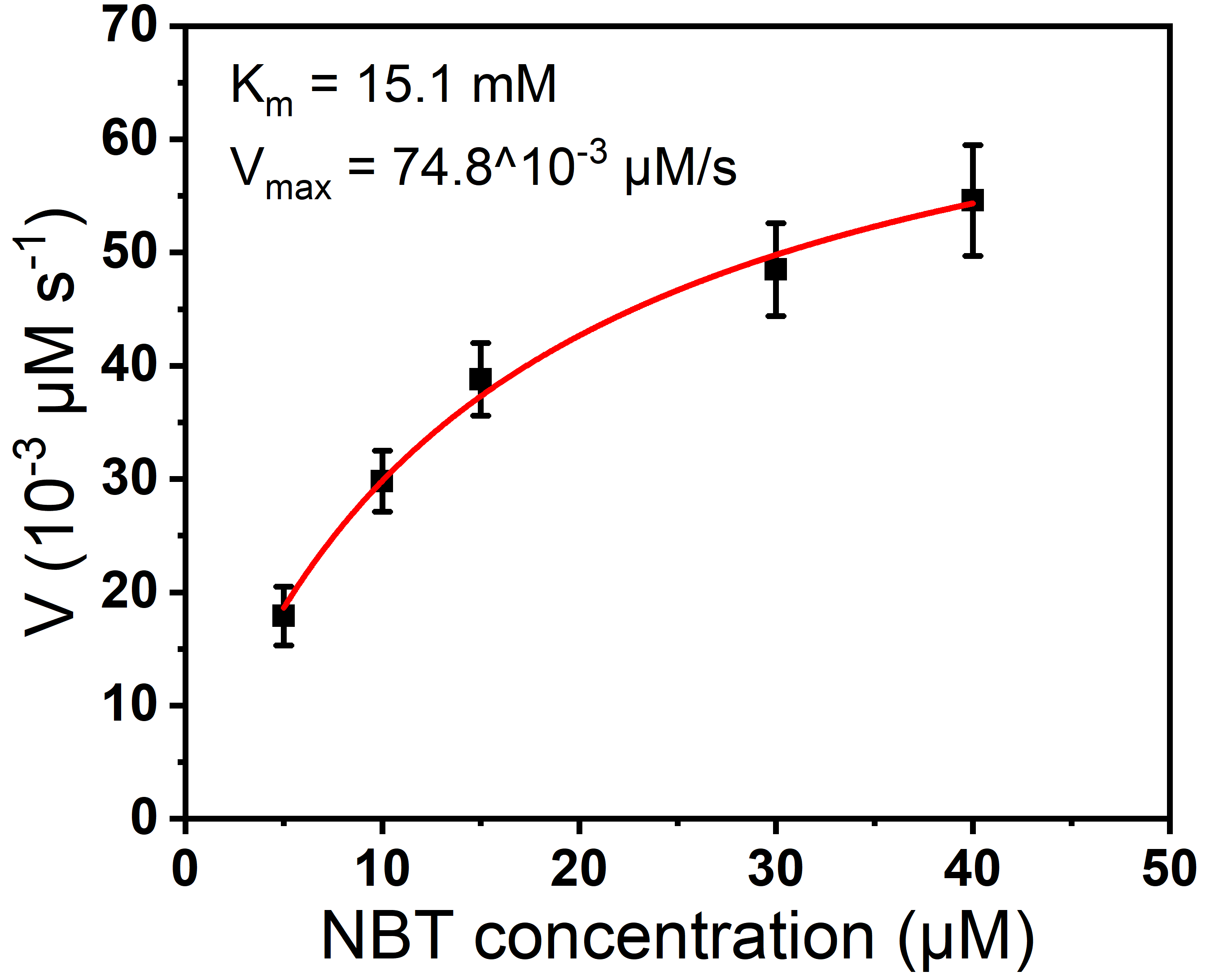


Fig. S8. The SOD-like activity of FCP NPs was evaluated by kinetic experiments.


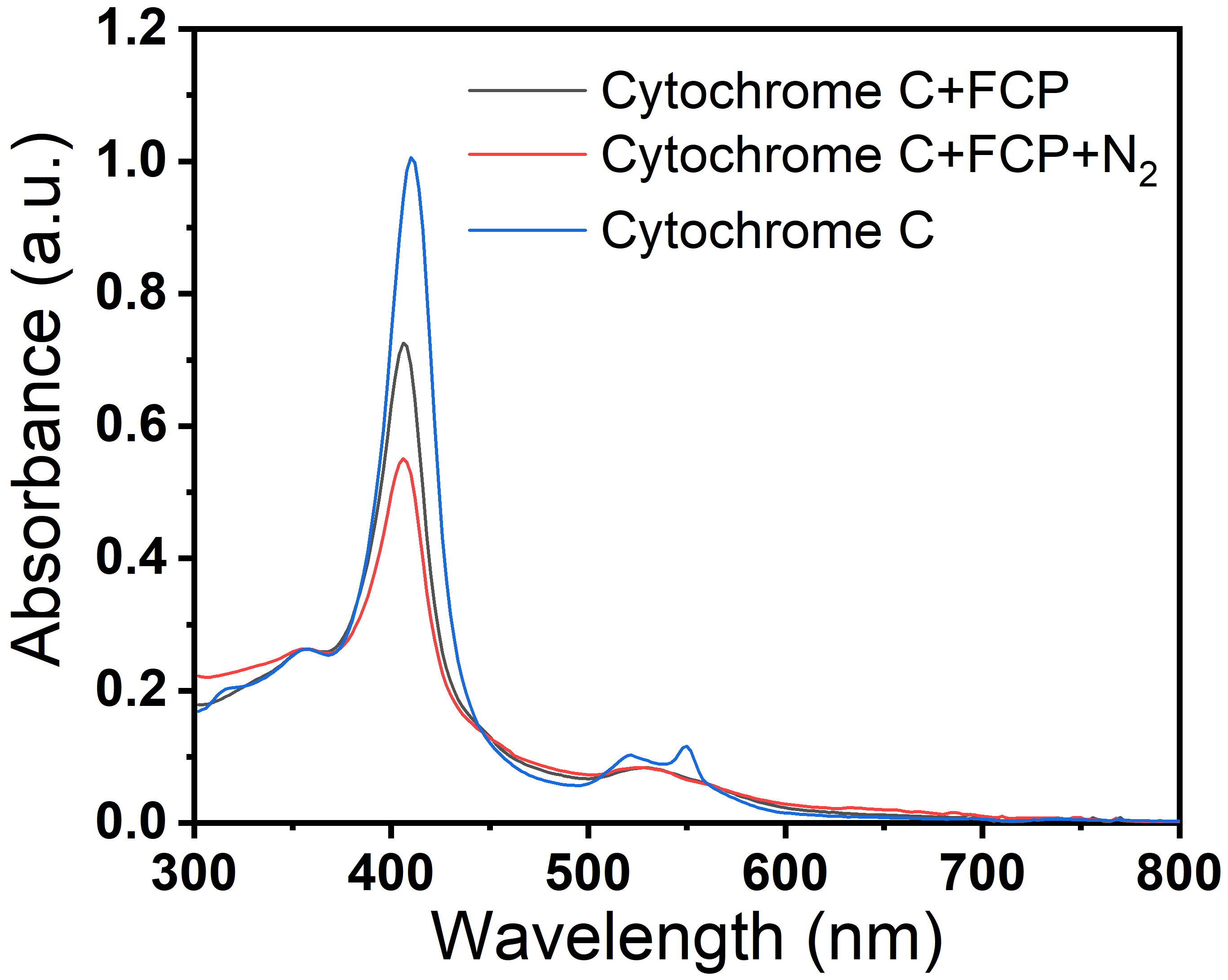


Fig. S9. Investigation of electron transfer between FCP and cytochrome C.


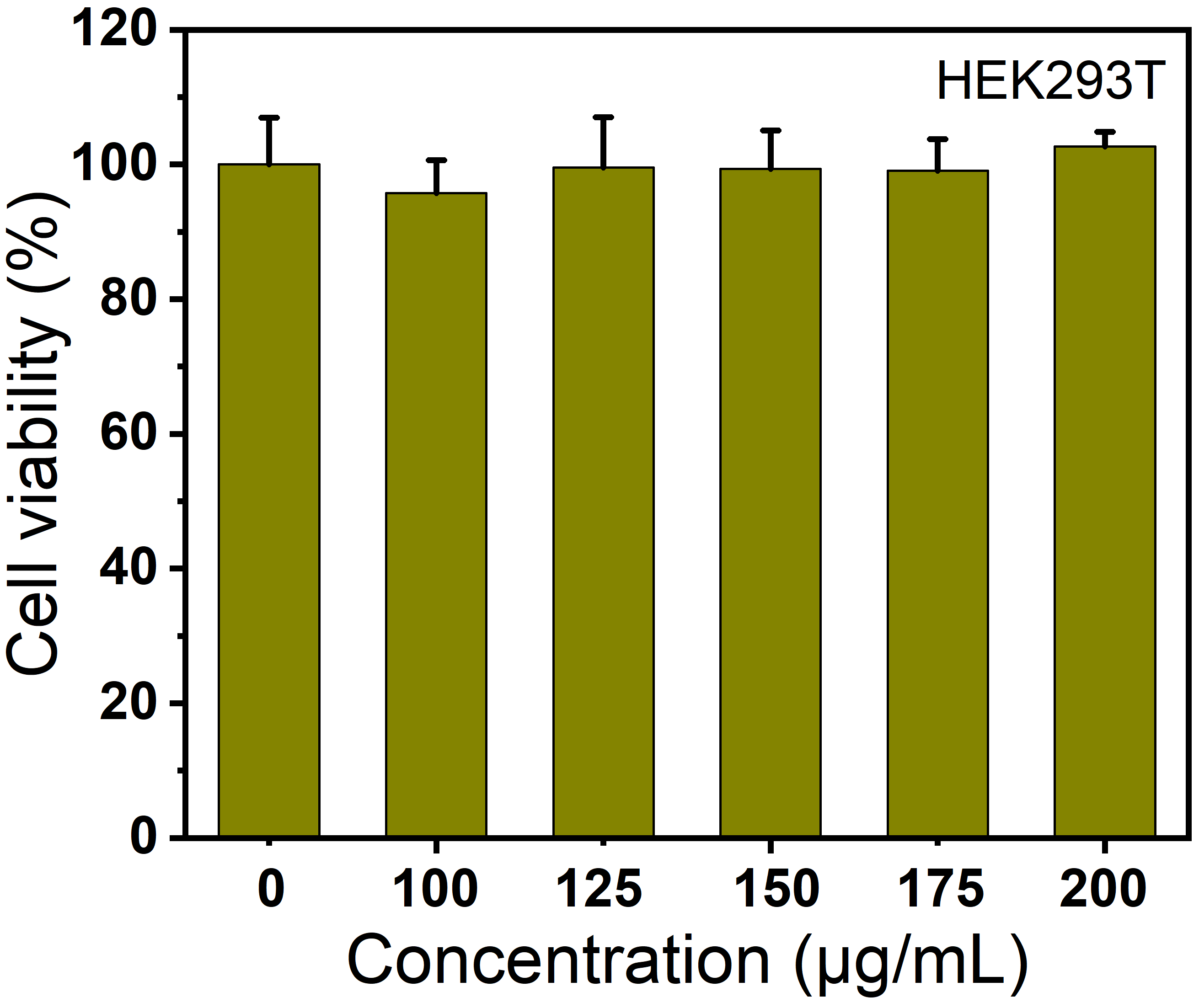


Fig. S10. Cell viability of HEK293T cells under different conditions as monitored by MTT assay.


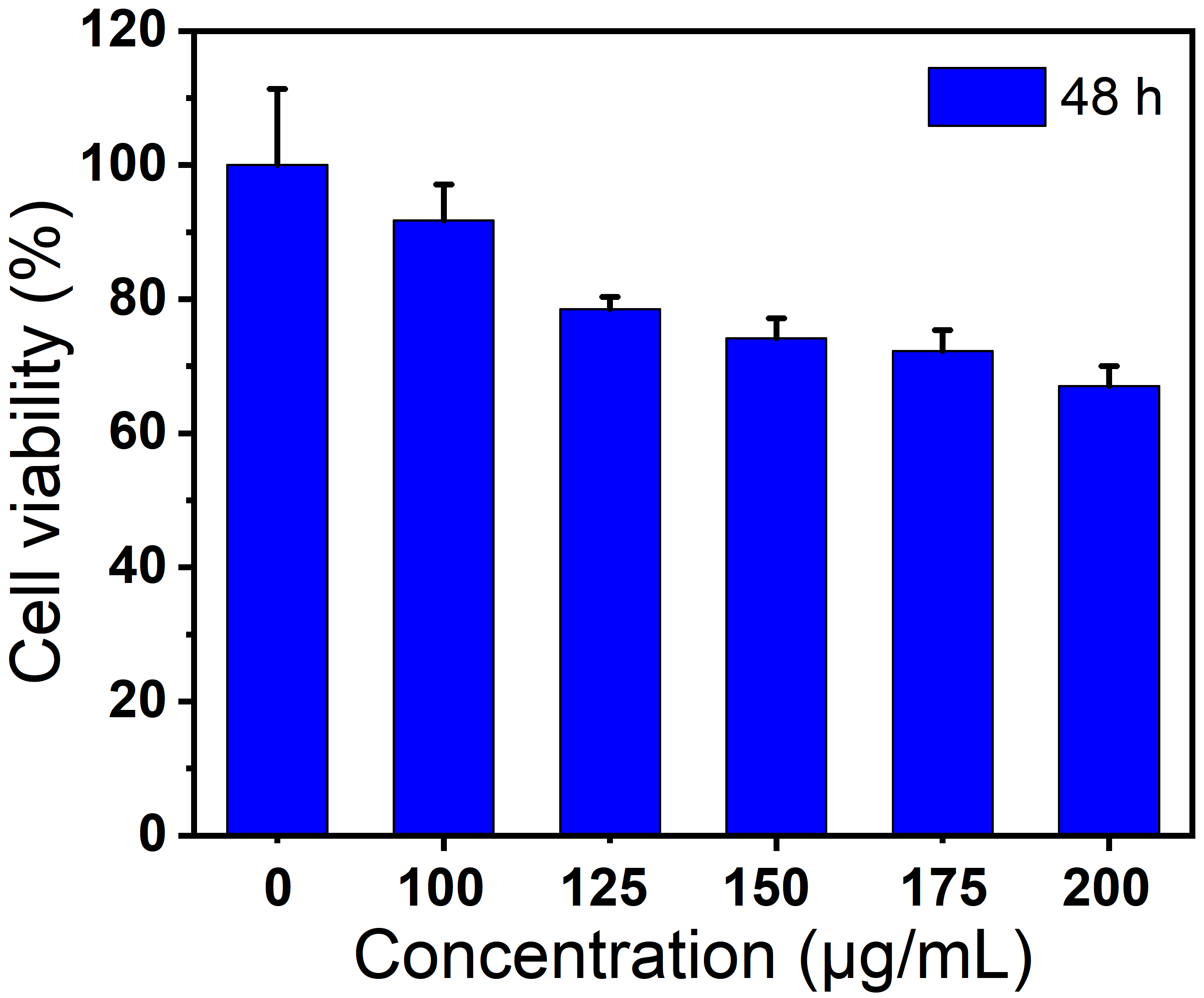


Fig. S11. Cell viability of 4T1 cells after incubation with FCP NPs for 48 h.


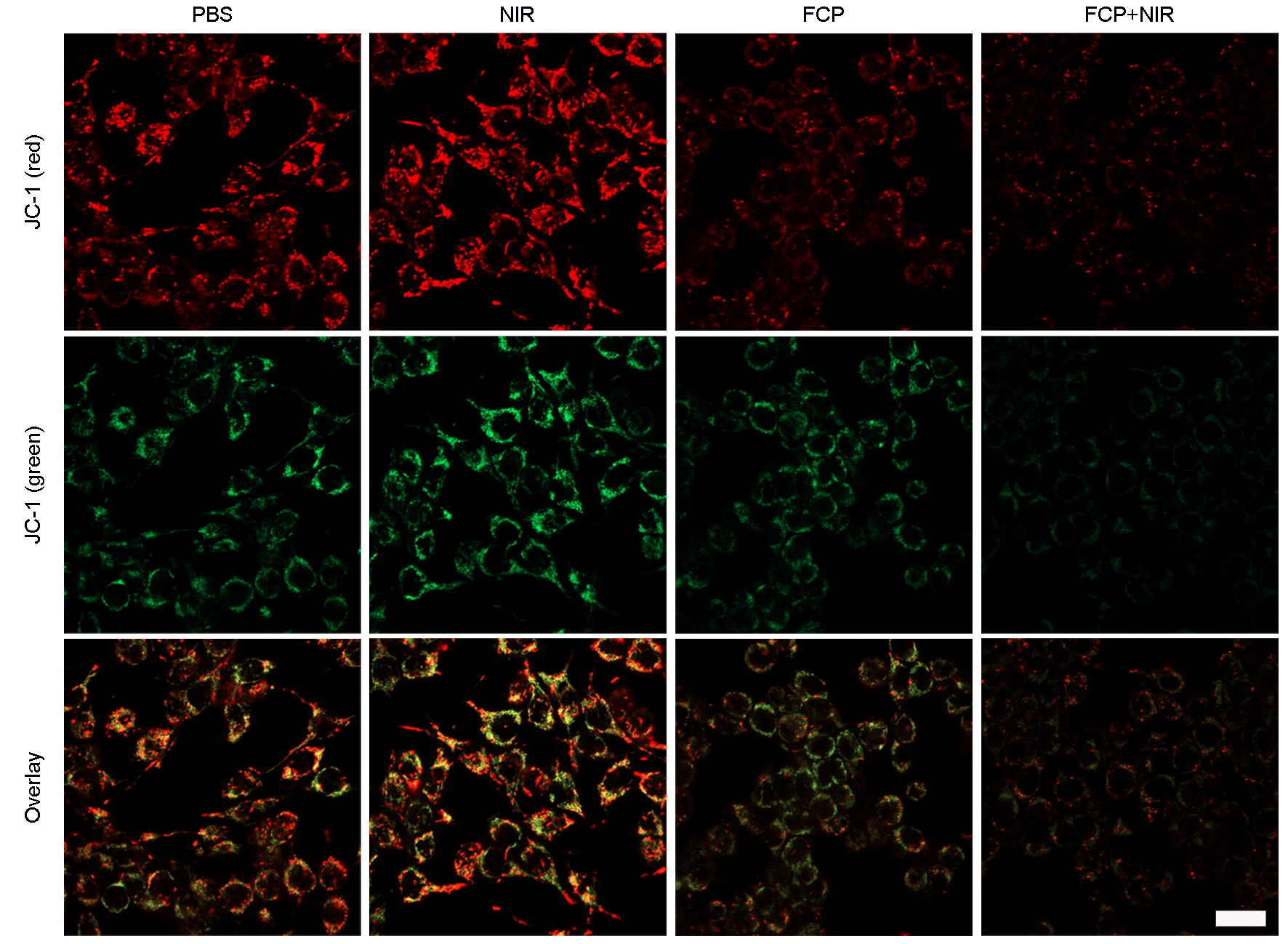


Fig. S12. Representative fluorescence images of 4T1 cells stained with JC-1 probe in different treatment groups. Scale bar: 20 μm.


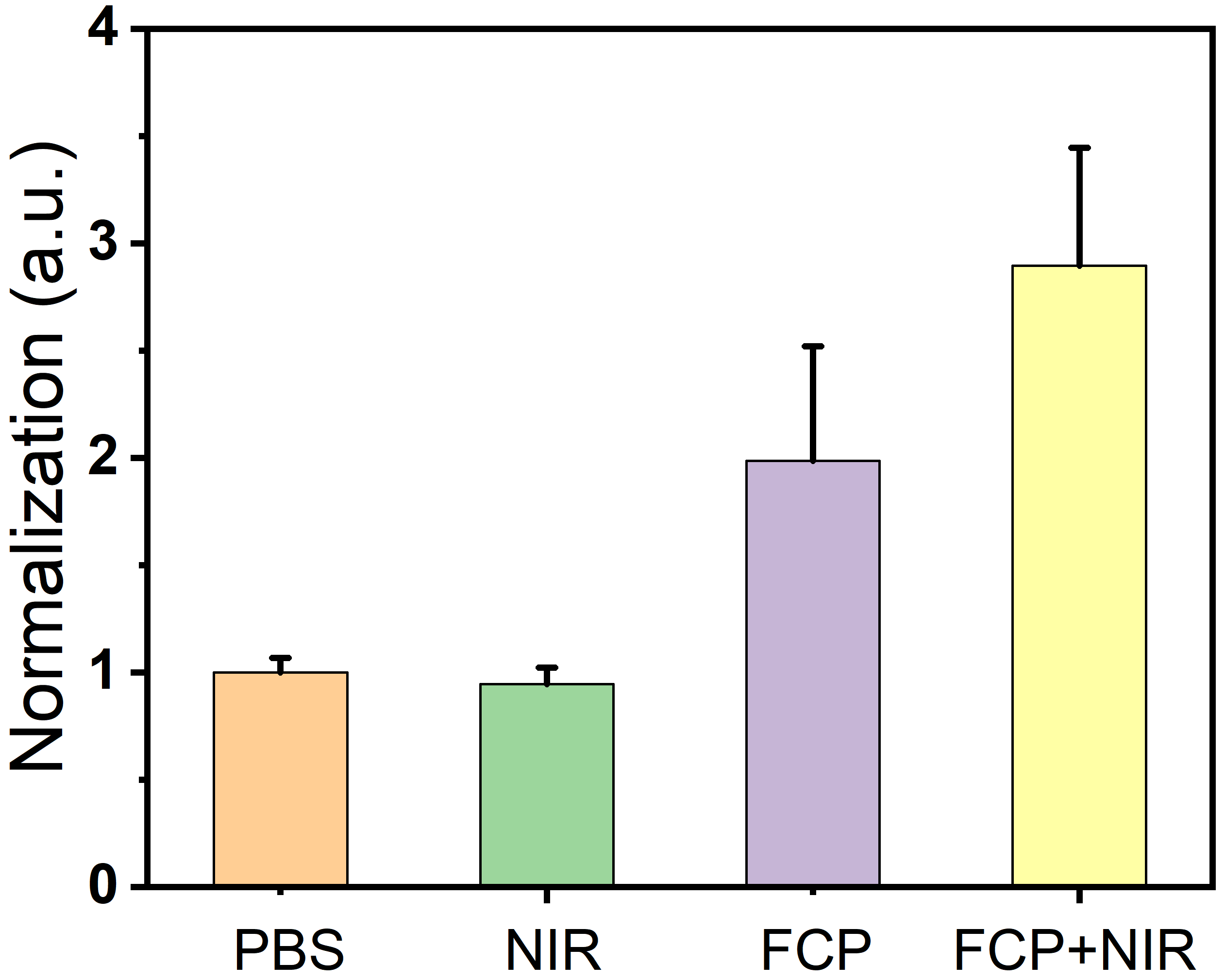


Fig. S13. The ratio of green fluorescence intensity to red fluorescence intensity in different treatment groups.


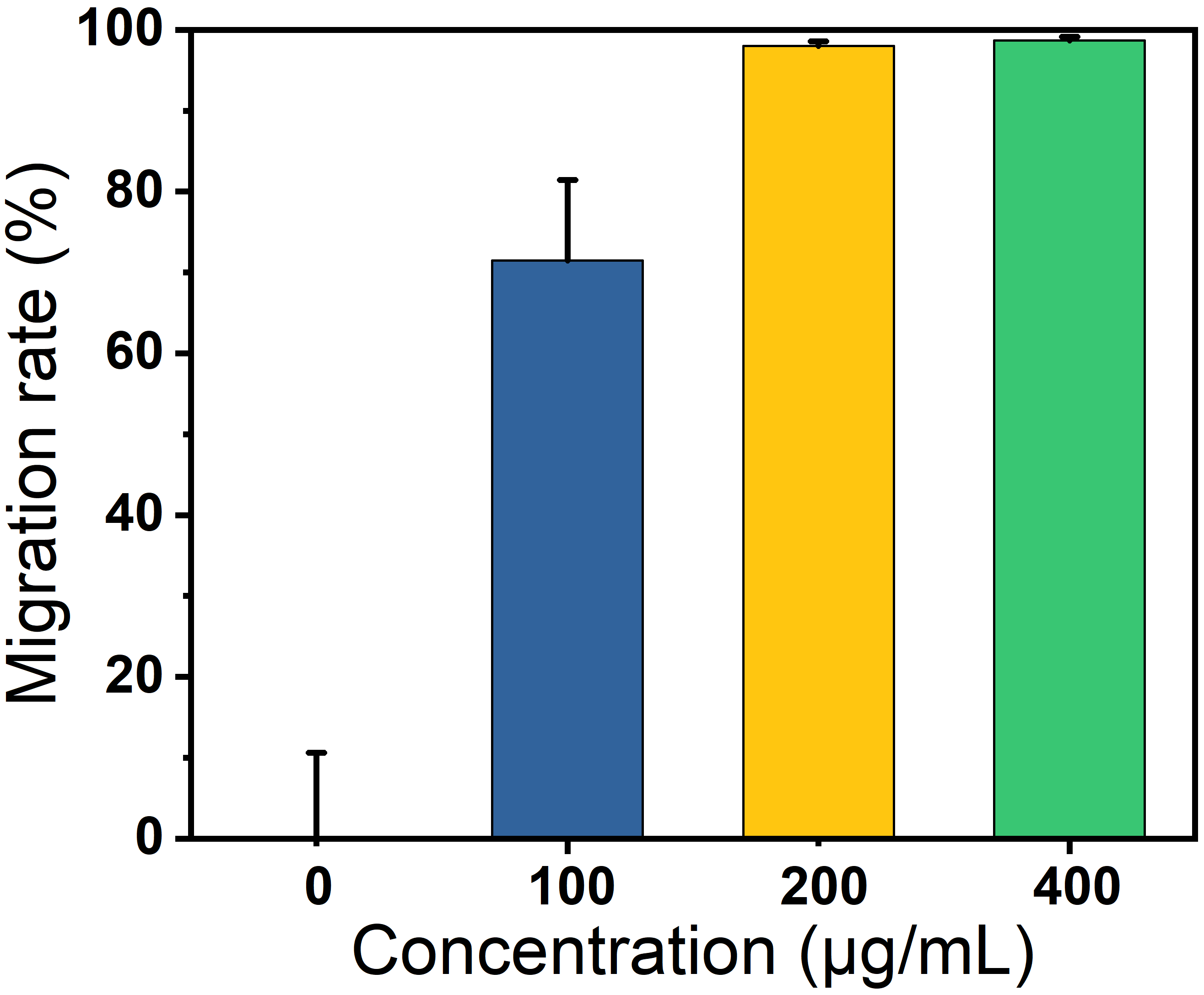


Fig. S14. Quantitative analysis of transwell assay under different conditions.


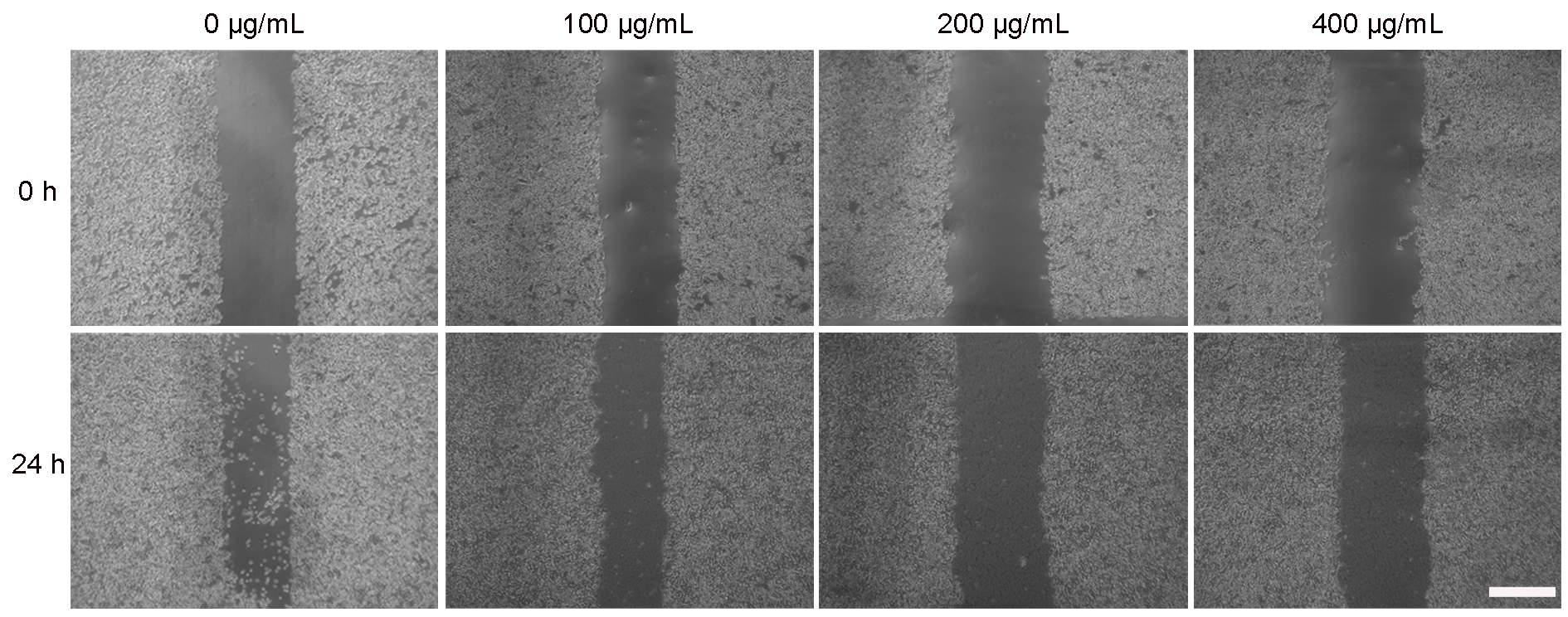


Fig. S15. Wound healing assay of 4T1 cells treated with indicated concentrations of FCP NPs. Scale bar: 500 μm.


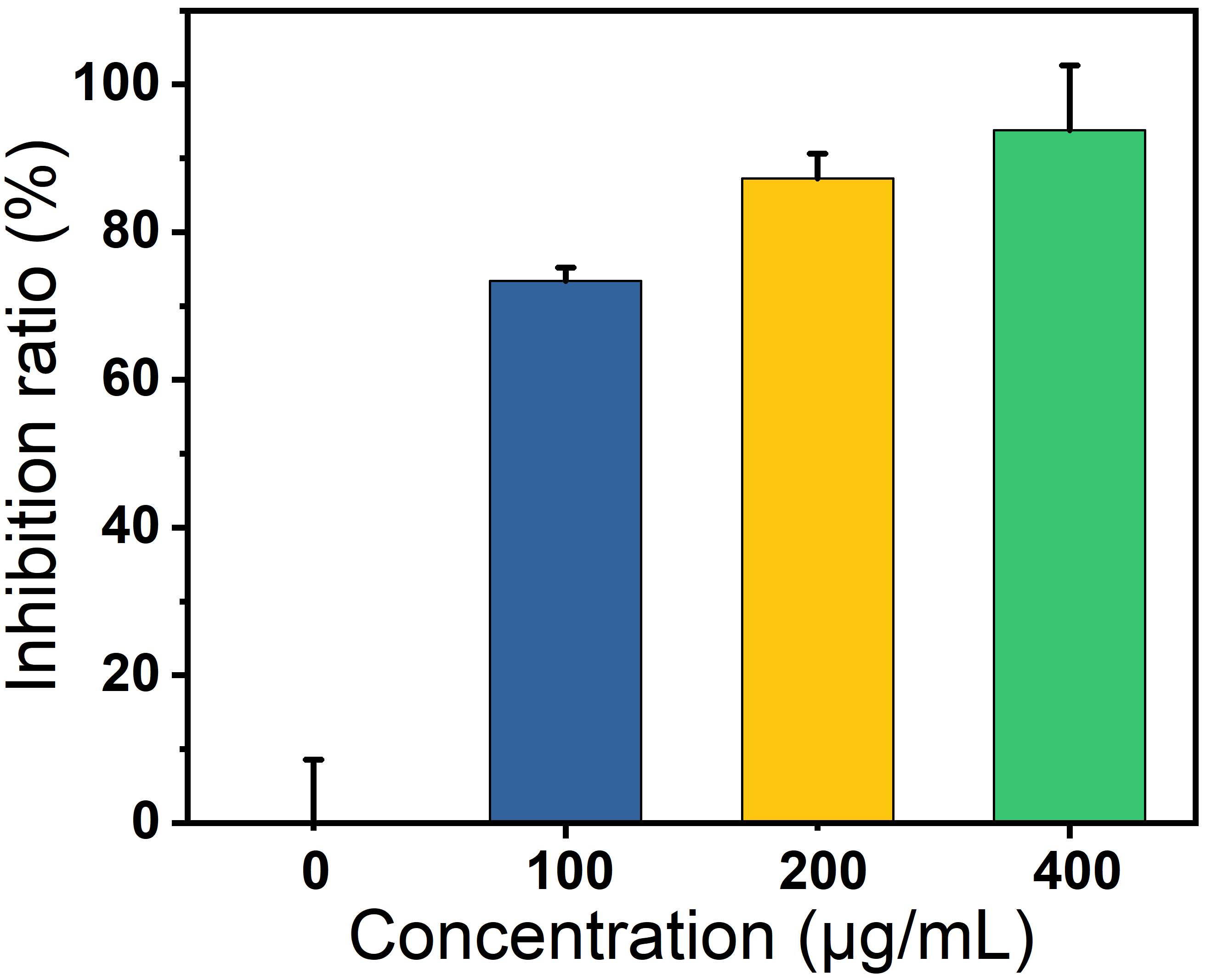


Fig. S16. Quantitative analysis of wound healing assay under different conditions.


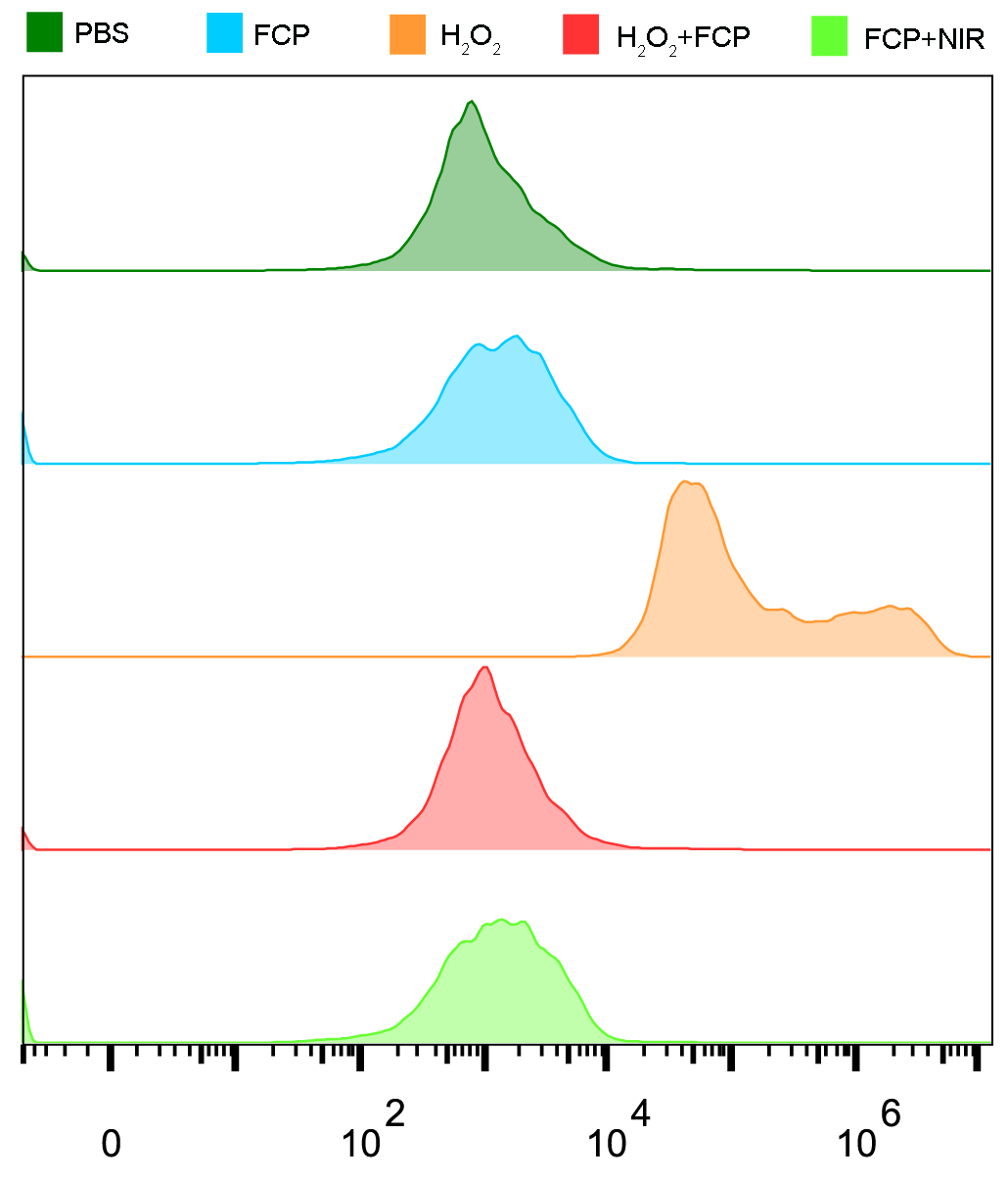


Fig. S17. [Flow cytometry](javascript:;) analysis of H_2_O_2_ stimulated 4T1 cells stained with DCF probe after pretreatment with/without FCP NPs.


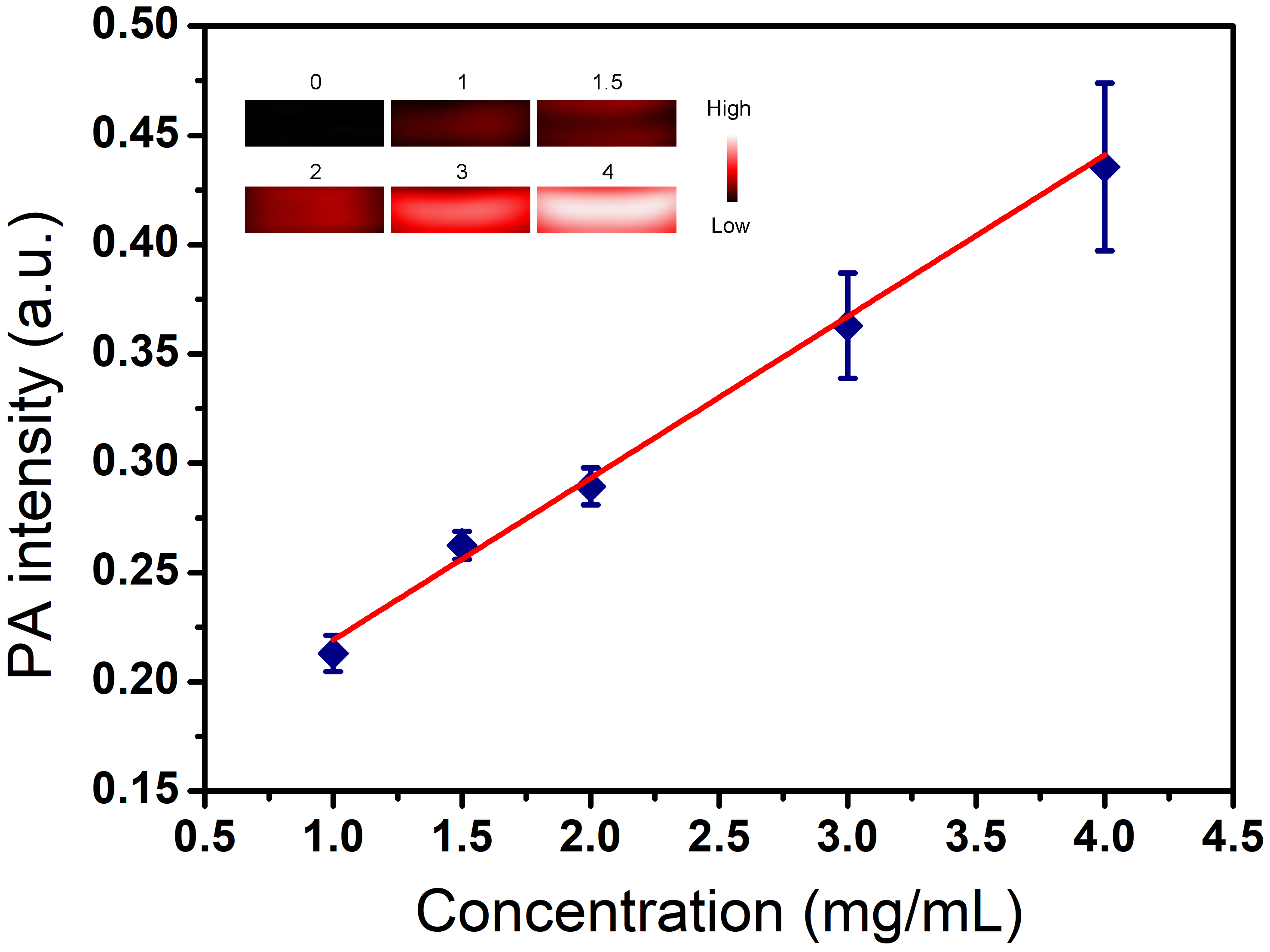


Fig. S18. PA imaging of FCP NPs dispersion with indicated concentrations.


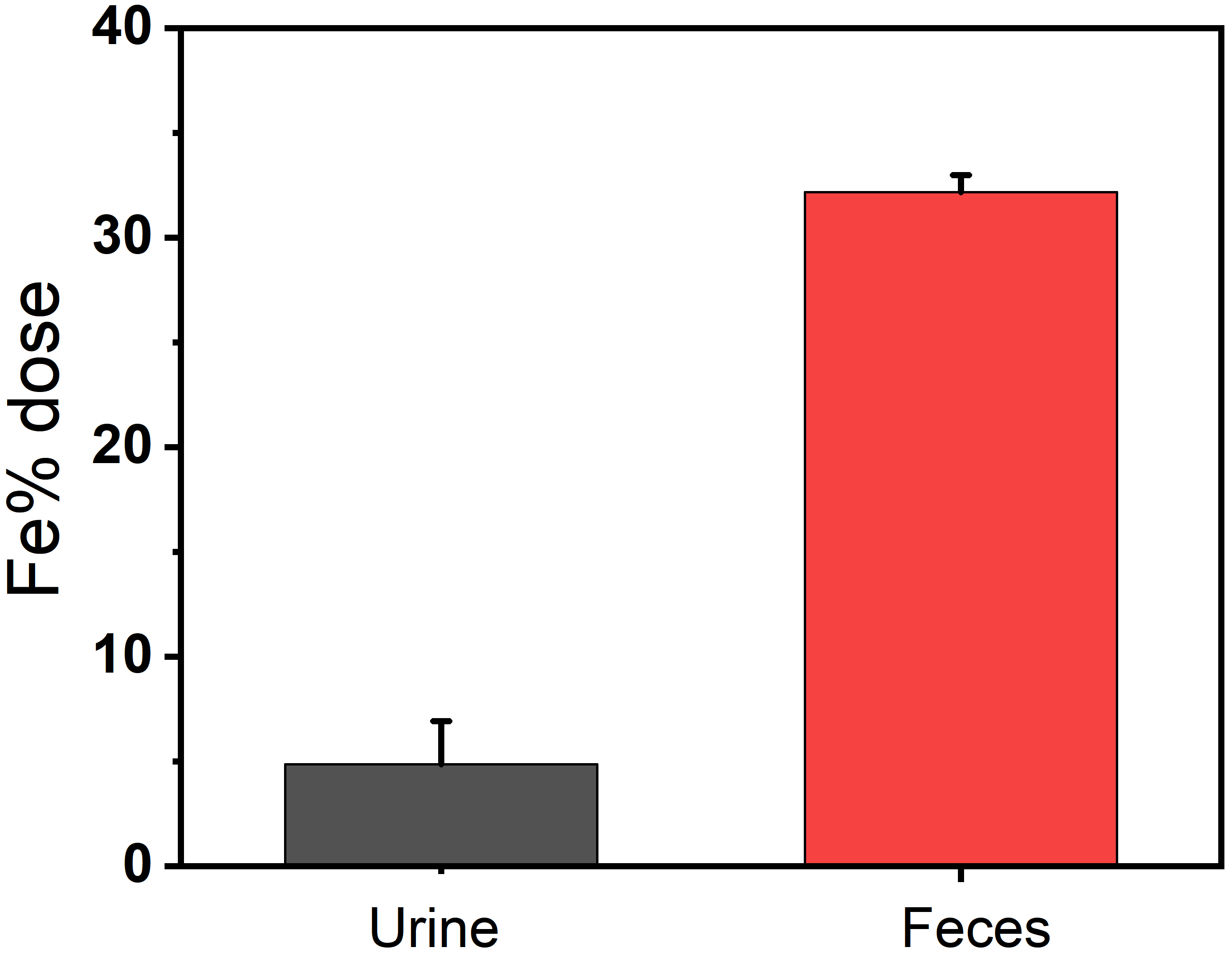


Fig. S19. The content of Fe in the feces and urine within 24 h post-injection of FCP as monitored by ICP-MS.


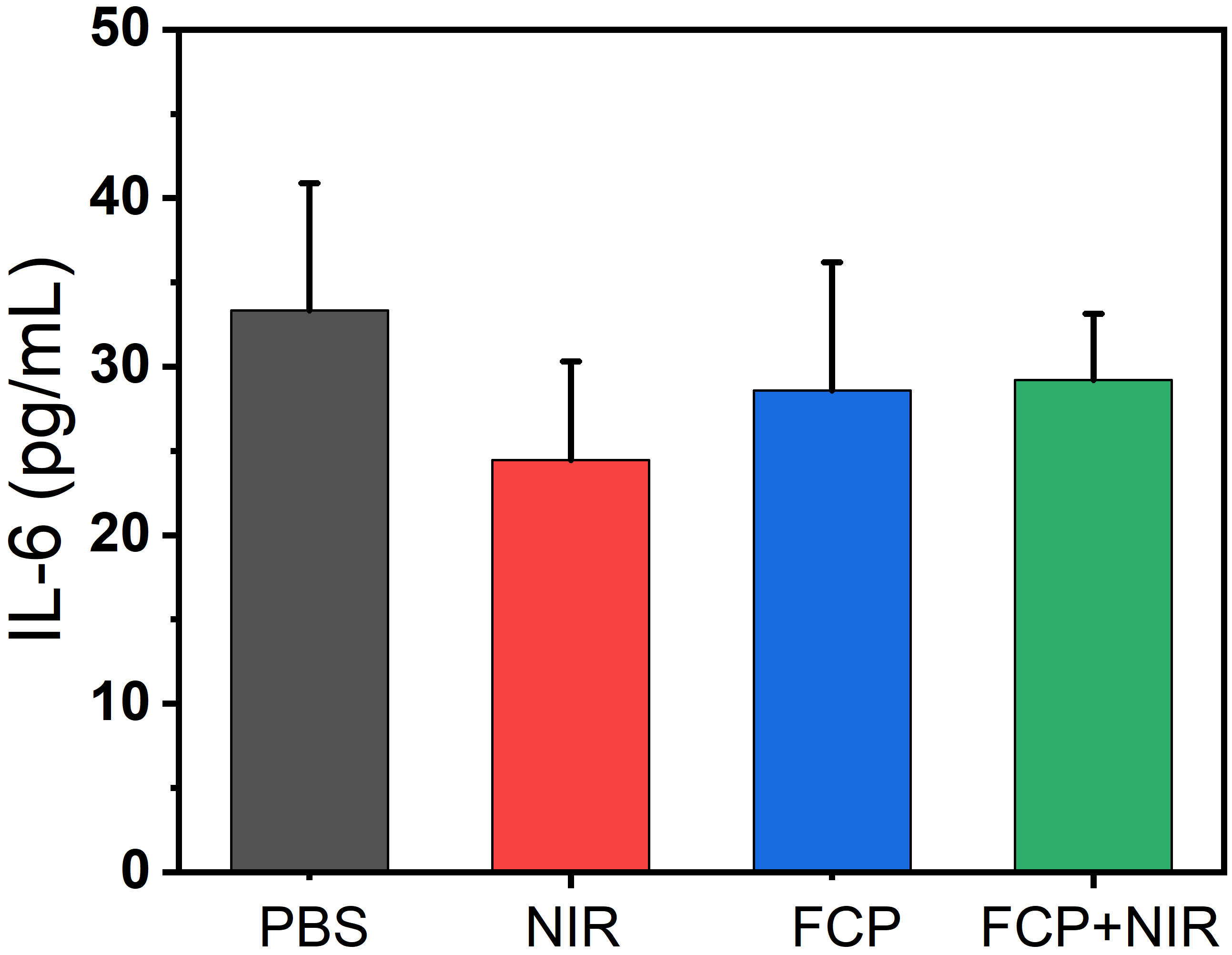


Fig. S20. The level of IL-6 in serum samples obtained from indicated groups.


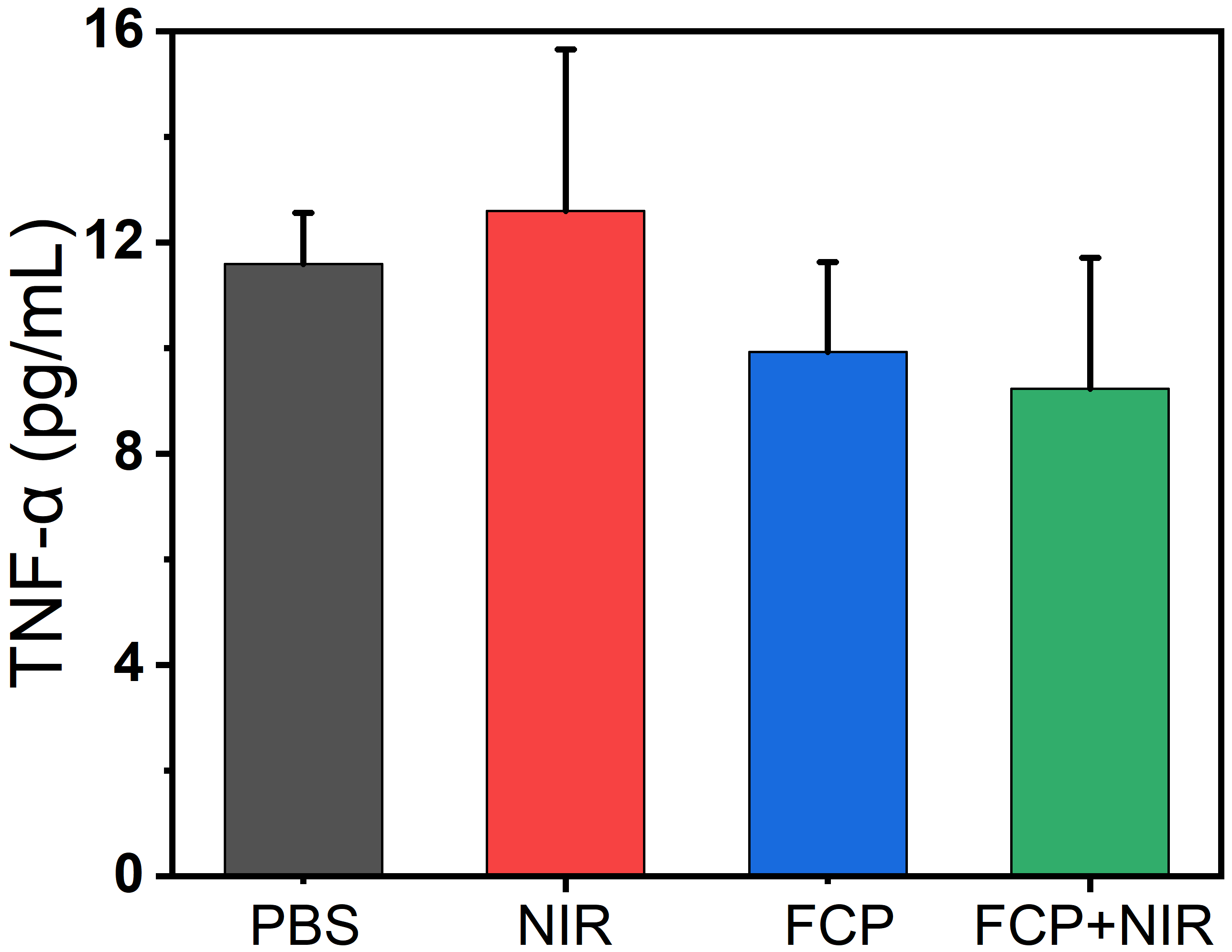


Fig. S21. The level of TNF-α in serum samples obtained from indicated groups.


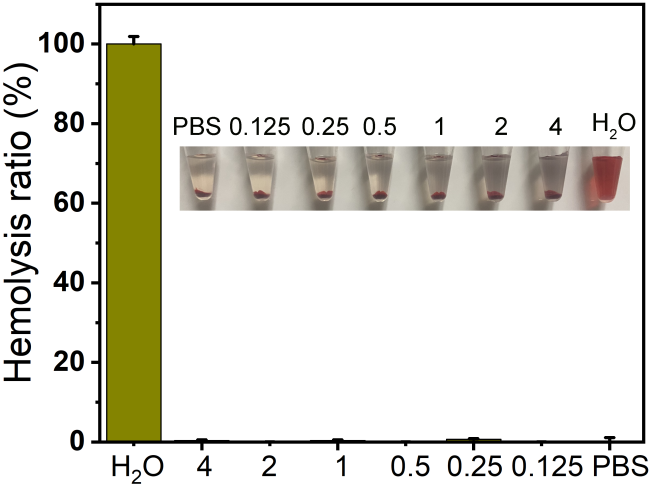


Fig. S22. Hemolytic test of FCP NPs with indicated concentrations.


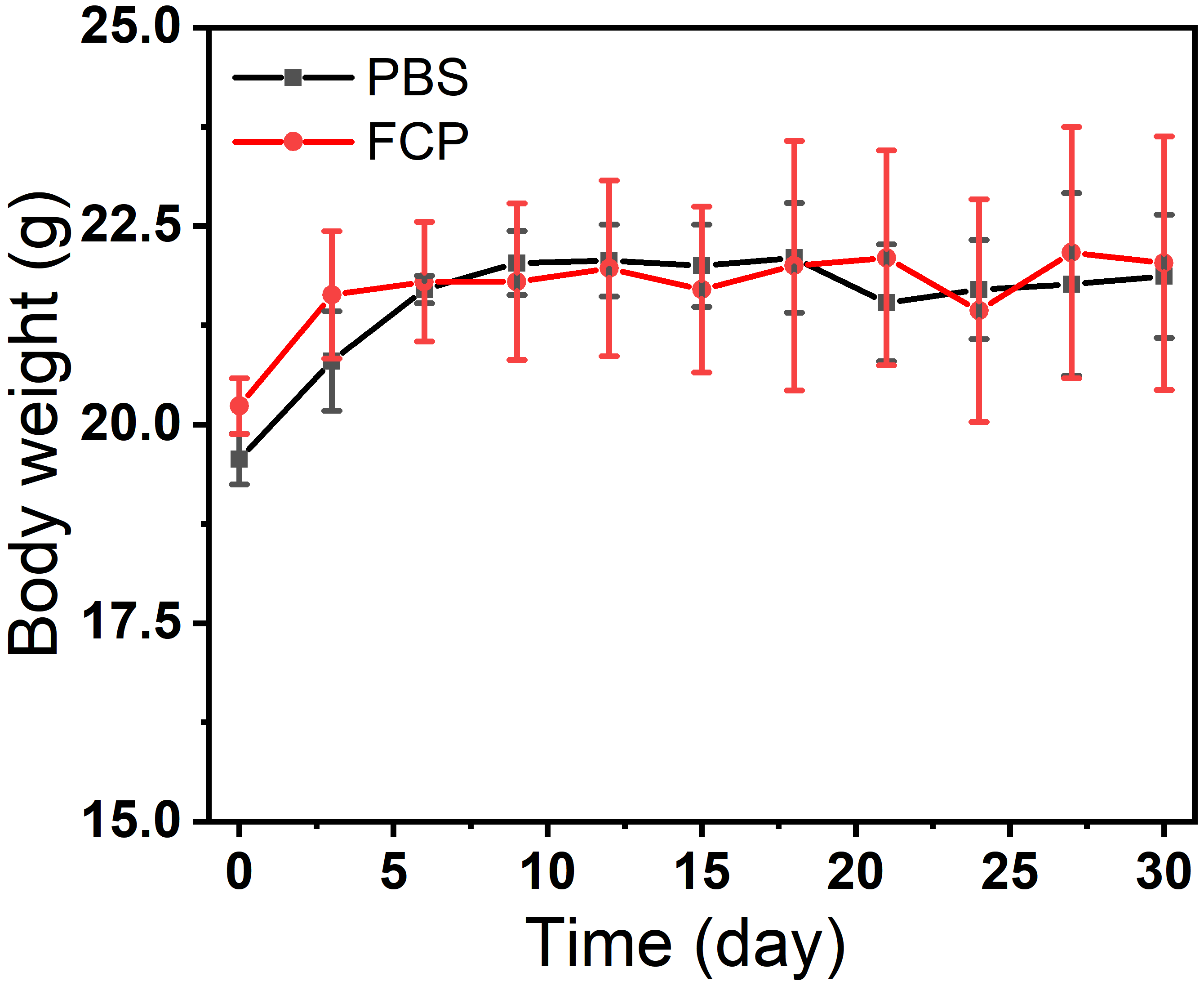


Fig. S23. Changes in the body weight of normal mice treated with PBS or FCP group for 30 days.
